# Polyamine metabolism patterns characterized tumor microenvironment, prognosis, and response to immunotherapy in colorectal cancer

**DOI:** 10.1186/s12935-023-02892-z

**Published:** 2023-05-18

**Authors:** Enkui Zhang, Chengsheng Ding, Shuchun Li, Batuer Aikemu, Xueliang Zhou, Xiaodong Fan, Jing Sun, Xiao Yang, Minhua Zheng

**Affiliations:** 1 Department of General Surgery, Ruijin Hospital, Shanghai Jiao Tong University School of Medicine, 197 Ruijin Er Road, Shanghai, 200025 China; 2 Shanghai Minimally Invasive Surgery Center, Ruijin Hospital, Shanghai Jiao Tong University School of Medicine, 197 Ruijin Er Road, Shanghai, 200025 China; 3 Department of General Surgery & Carson International Cancer Research Center, Shenzhen University General Hospital and Shenzhen University Clinical Medical Academy, Shenzhen, 518055 China

**Keywords:** Colorectal cancer, Polyamine metabolism, Tumor microenvironment, Immunotherapy, Prediction

## Abstract

**Background:**

Changes in Polyamine metabolism (PAM) have been shown to establish a suppressive tumor microenvironment (TME) and substantially influence the progression of cancer in the recent studies. However, newly emerging data have still been unable to fully illuminate the specific effects of PAM in human cancers. Here, we analyzed the expression profiles and clinical relevance of PAM genes in colorectal cancer (CRC).

**Methods:**

Based on unsupervised consensus clustering and principal component analysis (PCA) algorithm, we designed a scoring model to evaluate the prognosis of CRC patients and characterize the TME immune profiles, with related independent immunohistochemical validation cohort. Through comparative profiling of cell communities defined by single cell sequencing data, we identified the distinct characteristics of polyamine metabolism in the TME of CRC.

**Results:**

Three PAM patterns with distinct prognosis and TME features were recognized from 1224 CRC samples. Moreover, CRC patients could be divided into high- and low-PAMscore subgroups by PCA-based scoring system. High PAMscore subgroup were associated to more advanced stage, higher infiltration level of immunosuppressive cells, and unfavorable prognosis. These results were also validated in CRC samples from other public CRC datasets and our own cohort, which suggested PAM genes were ideal biomarkers for predicting CRC prognosis. Notably, PAMscore also corelated with microsatellite instability-high (MSI-H) status, higher tumor mutational burden (TMB), and increased immune checkpoint gene expression, implying a potential role of PAM genes in regulating response to immunotherapy. To further confirm above results, we demonstrated a high-resolution landscape of TME and cell–cell communication network in different PAM patterns using single cell sequencing data and found that polyamine metabolism affected the communication between cancer cells and several immune cells such as T cells, B cells and myeloid cells.

**Conclusion:**

In total, our findings highlighted the significance of polyamine metabolism in shaping the TME and predicting the prognosis of CRC patients, providing novel strategies for immunotherapy and the targeting polyamine metabolites.

**Supplementary Information:**

The online version contains supplementary material available at 10.1186/s12935-023-02892-z.

## Introduction

CRC is one of the most common malignancies and the second leading cause of cancer mortality. While surgery-based comprehensive treatment remains the conventional approach to treating CRC, it requires ongoing improvement [1]. Clinically, carcinoembryonic antigen (CEA) and imaging are the main detecting strategies for CRC screening, surgical evaluation, and recurrence and metastasis of CRC patients [2, 3]. However, improving the sensitivity and specificity of these methods necessitates the development of more accurate predictive models and biomarkers.

With the in-depth exploration of the relationship between the immune system and cancer, immunotherapy targeting programmed cell death 1 (PD1)/programmed cell death-ligand 1 (PD-L1) and cytotoxic T-lymphocyte antigen 4 (CTLA4) has revolutionized tumor treatment [4]. Immunotherapy has shown a certain therapeutic response in several malignancies, including melanoma, bladder cancer, non-small cell lung cancer, mismatch repair tumors (MMR), and MSI colorectal cancer [5–8]. These clinical studies have spurred research on tumor immunity, of which the tumor microenvironment (TME) is the main representative. Assessing the degree of immune cell infiltration in tumors based on the characteristics of TME is an important research tool to predict patient prognosis and response to immunotherapy. Consequently, prognostic models consisting of immune checkpoints and other factor are becoming auxiliary tools for clinical diagnosis and treatment [9–11]. Developing novel therapeutic strategies aimed at mobilizing the immune system to eradicate tumor cells will advance the field of immunotherapy for promote its effectiveness in cancer treatment.

Metabolic reprogramming of cancer cells is the leading directions for tumorigenesis and disease development. Tumor cell metabolism plays a key role in driving tumor survival, progression, immune evasion, drug resistance, and disease recurrence [12]. It is generally believed that the accumulation of abnormal metabolites can promote the occurrence and development of tumors, and closely related to tumor heterogeneity, which also affects the tumor microenvironment and the enrichment and activity of immune cells [13]. Targeting tumor cell metabolism has been shown to provide patients with survival benefits and enhance tumor sensitivity to therapeutic interventions [14]. Among the various metabolic pathways, polyamine metabolism (PAM) has been found to be closely related to cancer cells in recent studies [15–17].

Polyamines, including putrescine, spermidine and spermine, are important cationic alkylamines involved in various cellular processes essential for normal cell growth [18]. For cancer, PAM is usually dysregulated, and research on this as a therapeutic target emerges in an endless stream, so PAM has long been considered a potential cancer treatment direction [19]. PAM predicts the activation of tumor metabolic reprogramming, which is closely related to the occurrence and development of tumors. ODC1, a key enzyme of PAM, is the core rate-limiting enzyme of PAM, and the proteasome family genes is the basis for amino acid synthesis of polyamine raw materials, and jointly participates in the synthesis and metabolism of polyamines [20]. PAM enhancement is often indicative of continuous cancer cells proliferation. Modern views have also confirmed that some oncogenes such as MYC, BRAF, and all contribute to the maintenance of PAM [21–23]. Research on the regulation of PAM in survival of CRC has attracted much attention. However, the mechanism of crosstalk between polyamines and TME remains unclear. Polyamines are thought to have anti-inflammatory and immunosuppressive properties, which suggesting that it is imperative to explore the association between polyamines and immune cells in the TME [24–26]. In order to investigate the role of PAM in CRC, we classified CRC samples into different PAM patterns (clusters). Subsequent analysis revealed there were different characterizations in cancer-related pathways and immune cell infiltration between clusters.

**Fig. 1:**
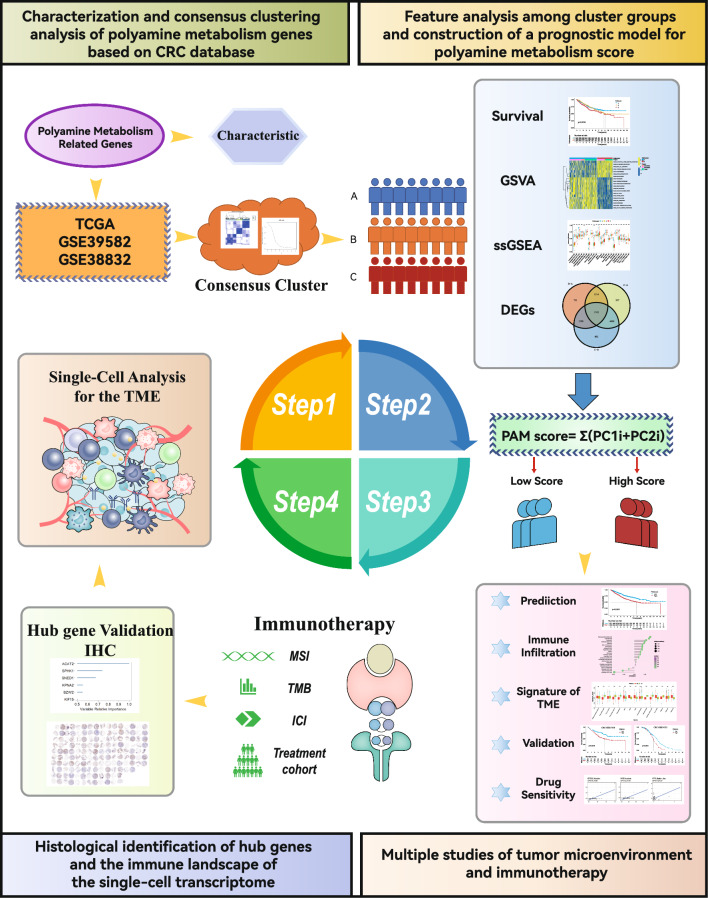
The workflow for constructing the pattern of polyamine metabolism genes of CRC survival prediction, immunotherapy, and immune landscape characteristics involved four steps. Firstly, we used PAM genes to identify relevant expression signatures in public databases and construct a consistent clustering model. Next, we conducted the corresponding difference analysis between the models. Based on this, we constructed a PAM scoring model for CRC survival prognosis. We performed a series of analyses of survival prognosis, tumor microenvironment, and immunotherapy between high and low subgroups of PAMscore and identified their signature genes. Finally, we delineated the immune landscape of genes involved in PAM based on single-cell data

We also constructed a PAM score model, which accurately predicts the prognosis of CRC patients and their response to immunotherapy. Furthermore, we conducted an extensive study of the TME and immunotherapy. Then, simple-cell RNA-seq (scRNA-seq) and metabolomics were currently the cutting-edge research methods to explore TME characteristics and mechanisms of metabolic reprogramming [27, 28]. Therefore, we analyzed the scRNA-seq dataset based on the constituent genes of this model to reveal the heterogeneity and metabolic features of the TME in PAM. Additionally, we demonstrated cellular metabolite communication in PAM. It further delineates the immune landscape associated with PAM. Our study presents a workflow for constructing a pattern of PAM-related genes for predicting CRC survival, immunotherapy response, and immune landscape characteristics (Fig. 1). This study demonstrated the TME characterization of different PAM patterns, providing a novel perspective for CRC research.

## Methods

### Collection of CRC data sources and preprocessing

Gene expression data and clinical information for CRC were obtained from the TCGA (https://portal.gdc.cancer.gov/) and NCBI GEO databases (https://www.ncbi.nlm.nih.gov/geo/). Specifically, the clinical data we used from TCGA mainly included age, gender, tumor stage, and overall survival (OS). In addition, four GEO CRC cohorts (GSE38832 [29], GSE39582 [30], GSE17536 [31], GSE14333 [32]) with GSE39582 clinical variables included age, sex, tumor stage, TP53, KRAS, BRAF and MMR mutation, follow-up time and survival status. RNA-sequencing data, including fragments per kilobase of transcript per million mapped reads (FPKM) values and counts, were consistently converted to transcripts per million (TPM) values. For microarray data from GEO, the normalized matrix files were directly downloaded and normalized using the “normalizeBetweenArrays” method of the R package “limma” after gene symbol conversion to ensure intensities or log ratios had similar distributions across a set of arrays. We merged three datasets (TCGA, GSE39582, GSE38832) and used the “ComBat” method from the 'SVA' R package to remove batch effects between different datasets [33].

Two immune checkpoint blockade treatment cohorts with available expression and clinical information were used in our study. The IMvigor210 cohort consisting of advanced transitional cell carcinoma of the urinary tract treated with the anti-PD-L1 antibody atezolizumab [34]. Besides, we obtained the Kim cohort consisting of metastatic gastric cancer treated with pembrolizumab [35].

### Single-cell RNA-seq data analysis

The “Seurat” R package (version 4.0.2) was used to perform scRNA-seq analysis [36]. GSE132465 [37] scRNA-seq cohort was utilized in the further visualization process through (Uniform Manifold Approximation and Projection) UMAP approach. The SingleR package was applied to annotate distinct cell clustering, and then different cell clusters were identified and annotated manually within the “CellMarker” database (http://biocc.hrbmu.edu.cn/CellMarker/) [38].

### Consensus clustering expression pattern of polyamine metabolism related genes

The genes in REACTOME_METABOLISM_OF_POLYAMINES were downloaded as Polyamine metabolism (PAM) genes from GSEA MSigDB. Overlapping PAM genes were identified between the TCGA and GEO cohorts. Based on these genes, consensus unsupervised cluster analysis was conducted to classify CRC samples into different PAM patterns using R package “ConsensusClusterPlus” [39].

### Gene set variation analysis (GSVA) and Gene ontology (GO) annotation

To elucidate the differences in biological processes between PAM patterns, we utilized R package "GSVA" to perform GSVA analysis on RNA-seq data of CRC samples and reference gene sets from “c2.cp.kegg.v7.5.1.symbols.gmt”, which was downloaded from MSigDB [40]. The GO and KEGG analysis were performed on genes related to PAM by R package “clusterProfiler”, with q < 0.05 as the filter condition [41, 42].

### Immune cell infiltration estimation by ssGSEA and deconvolution algorithm

We utilized single-sample gene set enrichment analysis (ssGSEA) to measure the relative abundance of 23 types of immune cell in the TME. The specific gene sets used to mark each immune cell type were picked from the past study [43]. The relative abundance of each immune cell type was represented by an enrichment score in the ssGSEA analysis and normalized to a uniform distribution ranging from 0 to 1.

### Construction of the PAMscore

In order to quantify the heterogeneity of each CRC sample, a scoring system was developed. We identified 1742 differentially expressed genes (DEGs) from different PAMclusters, and then performed a prognostic analysis on these genes using “limma” [44]. The significance criteria for determining DEGs were set as adjusted P values < 0.001, and the adjusted P value for multiple testing was calculated using the Benjamini–Hochberg correction.

Subsequently, we condcutde PCA analysis on these prognostic DEGs and extracted principal components 1 and 2 (PC1 and PC2) as feature scores. The method focuses on the score of the ensemble with the largest chunk of well-related (or inversely correlated) genes in the ensemble while reducing the contribution of genes that are not tracked with other ensemble members. We then adopted a formula similar to previous studies to define PAMscore while reducing the contribution of genes not tracked with other ensemble members [45, 46]. The specific formula is as follows: PAM score = ∑ (PC1i + PC2i).

### Prognostic analysis for high and low subgroup of PAMscore

We employed PCA analysis to determine the score of 1224 samples separately. After that, we used "Survminer" to select the best cut-off value, dividing all samples into high- and low-PAMscore subgroups, and performed survival analysis on the two subgroups.

### Correlation analysis of PAM score gene and drug sensitivity

We used the CellMiner database (https://discover.nci.nih.gov/cellminer/home.do) to download gene expression and drug sensitivity data for the same sample. We filtered drug sensitivity data after clinical laboratory verification and FDA standard certification [47]. Then we combined the 328 PAMscore-related gene expression with drug sensitivity data and performed the Pearson correlation test to obtain correlation relationship between PAMscore-related gene expression and drug sensitivity.

### Immunohistochemistry (IHC)

For the 328 model-building genes constructed by the PAM score, we used the random forest method to perform feature selection to obtain 6 representative feature genes. Six proteins ACAT2, SPHK1, SNED1, KPNA2, BZW2 and KIF15 were identified by IHC using tissue microarray (TMA). The TMA we used contained 75 colorectal cancer and paired adjacent normal paraffin-embedded specimens, provided by the Shanghai Minimally Invasive Surgery Center of Ruijin Hospital (Shanghai, China) in accordance with the guidelines of the Ethics Committee of Ruijin Hospital. All cases signed written informed consent before the study.

We baked the paraffin sections at 62 °C for 1 h, dewaxed, heated antigen retrieval, added primary antibody after washing, and kept overnight at 4 °C. ACAT2 (1:100 dilution ratio), KPNA2 (1:100 dilution ratio), KIF15 (1:200 dilution ratio) and SPHK1 (1:50 dilution ratio) were purchased from Proteintech (14755-1-ap, 10819 -1-AP, 55407-1-AP, 10670-1-AP), SNED1 (1:100 dilution ratio) were purchased from Sigma (HPA036414), BZW2 (1:100 dilution ratio) were purchased from Signalway (46935). The next day, incubate with horseradish peroxidase (HRP)-conjugated secondary antibody for 30 min at 37 °C. Finally, the neutral gum was mounted, and a coverslip was added. Following immunohistochemical assays, two independent pathologists analyzed the expression levels of target proteins. By cell staining ratio (0 = 0%, 1 ≤ 25%, 2 = 26 to 50%, 3 = 51 to 75%, 4 = 76 to 100% positive), open to judge the expression of the target protein.

### Statistical analysis

The Statistical analyses in this study were generated by R-4.1.2. We estimated statistical significance of normally distributed variables for quantitative data by Student's t-test, and Wilcoxon test and Kruskal–Wallis test for nonparametric or parametric methods for comparison [48]. Correlation coefficients were calculated using Spearman’s and distance correlation analyses. Receiver operating characteristic (ROC) curves were used to verify the validity of the model. For all survival analyses, we used Kaplan–Meier survival analysis and Cox proportional hazards model analysis in the R package "Survminer" for prediction and repeated testing of all potential cut points to select the best cutoff point. In this study, all statistical analyses were two-sided and statistical significance was set at P < 0.05.

## Results

### Characteristics and cluster analysis of polyamine metabolism genes in colorectal cancer

59 PAM-related genes were obtained from REACTOME_METABOLISM_OF_POLYAMINES in the GSEA MsigDB database (Additional file 1: Table S1). We then conducted a differential analysis of the RNA expression levels of these 59 genes in normal tissues and tumor tissues in the TCGA database, and found that the expression of most PAM-related genes were significantly different between colon cancer and normal colon samples (Additional file 2: Fig. S1A). We also employed heatmap of significantly different PAM genes to show RNA expression between normal tissues and tumors (Additional file 2: Fig. S1B). GO and KEGG enrichment analysis demonstrated PAM genes were enriched in the pathways related to PAM, protein or amino acid metabolism and the synthesis of enzymes (Additional file 2: Fig. S1C, D). we also showed the protein–protein interaction (PPI) network of the above 59 genes, and found that proteasome family genes and ODC1 played a major role in the network (Additional file 2: Fig. S1E). Besides, we performed a survival analysis of the above PAM related genes using Kaplan–Meier method and listed genes with significant differences (Additional file 2: Fig. S2).

**Fig. 2:**
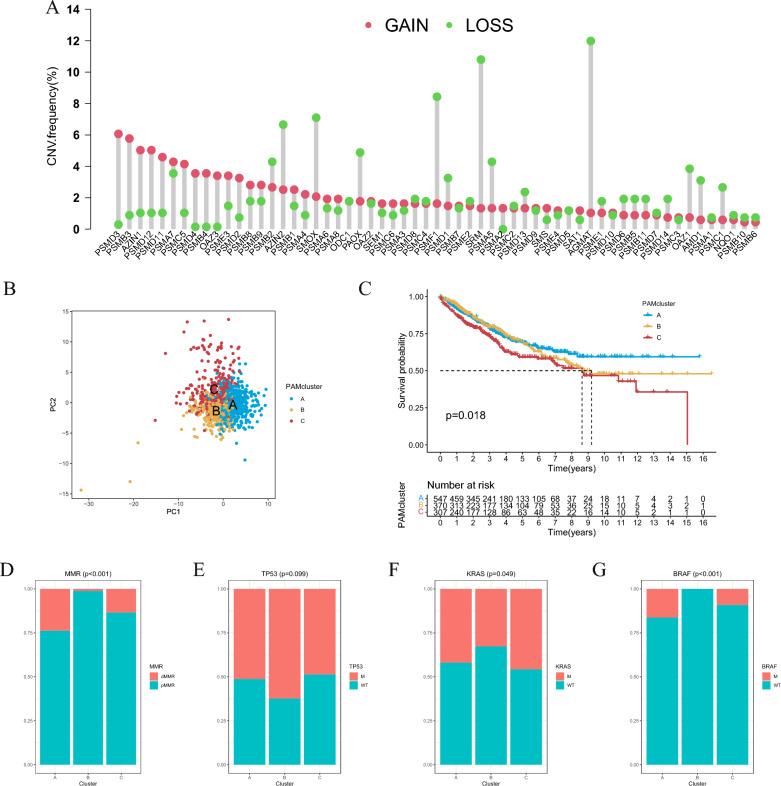
**A** The CNV mutation frequency of 59 polyamine metabolism (PAM) related genes was prevalent. The column represented the alteration frequency. The deletion frequency, green dot; The amplification frequency, red dot. **B** Kaplan–Meier curves of overall survival (OS) for 1224 CRC patients in meta cohort with different PAM clusters. The numbers of patients in PAMcluster-A, PAMcluster-B, and PAMcluster-C phenotypes are 547, 370 and 307, respectively (Log-rank test). **C** PCA analysis showing a remarkable difference in transcriptomes among the three clusters. **D**–**G** The proportion of mutation features in the three patterns based on GES39582

Next, we also examined the somatic copy number variation (CNV) of the above 59 genes and found that CNV gain was the most prevalent change, which might account for the high expression of PAM genes in cancer tissues. Strong CNV deletion was observed in several genes such as AZIN2, SMOX, PAOX, PSMF1, PSMD1, SRM, PSMA5, PSMB2, AGAMT, OAZ1, AMD1 and PSMC1, which also showed decreased in gene expression (Fig. 2A). Afterwards, we took the intersection of common PAM-related genes in TCGA, GSE39582 and GSE38832, and finally obtained 55 PAM-related genes (Additional file 2: Fig. S1G). First, PCA analysis revealed clear boundaries between three PAM patterns, suggesting that CRC samples of different PAM patterns may have different characteristics. (Fig. 2B). In order to explore the prognostic value of PAM-related genes and the mechanisms for the tumorigenesis and progression of CRC, 1224 CRC samples from TCGA, GSE39582, and GSE38832 were classified into 3 clusters by unsupervised consistent clustering (Additional file 2: Fig. S3). There were 547 in pattern A, 370 in pattern B, and 307 in pattern C (Additional file 1: Table S2), which were defined as PAMclusterA, B and C. PAMcluster-A showed significant better survival, while PAMcluster-C had the worst prognosis (Fig. 2C). In terms of clinical characteristics, the proportion of gene mutations in different patterns was explored. We found that in the comparison of the results of MMR, KRAS and BRAF mutations, the proportion of patients with mutations in PAMcluster-A was generally higher than that in PAMcluster-B and PAMcluster-C (Fig. 2D–G).

### Analysis of differential pathways and immune cell infiltration

**Fig. 3:**
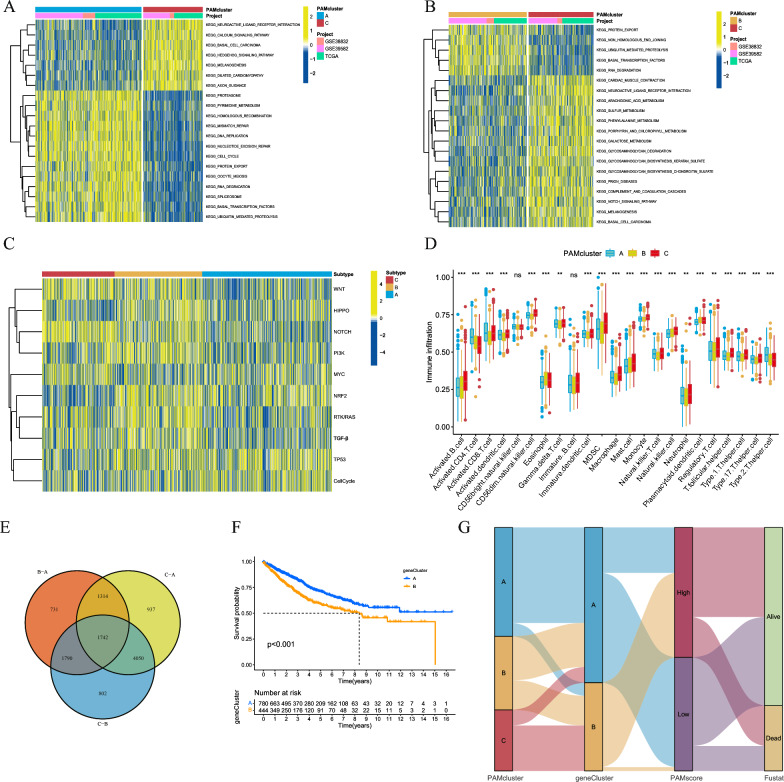
**A**, **B** GSVA enrichment analysis demonstrates different KEGG biological pathways among three distinct clusters, in which yellow and blue represent activated and inhibited pathways, respectively. **C** Heatmap for differential analysis of cancer-related signaling pathways in three clusters. **D** Boxplot of abundance of immune cells in three clusters based on the RNA-seq meta cohort. **E** Venn diagram has shown polyamine metabolism related differentially expressed genes (DEGs) between three PAM cluster. **F** Kaplan–Meier curves of overall survival (OS) for 1224 CRC patients in meta cohort with different geneCluster. **G** Sankey diagram demonstrates the changes of PAMcluster, geneCluster, PAMscore and survival status. (*P < 0.05; **P < 0.01; ***P < 0.001; ns, non-significant)

GSVA showed that PAMcluster-C was enriched with various nutrient metabolism pathways, including amine metabolism, when compared to PAMcluster-B. In contrast, pathways related to mismatch repair and cell repair were not enriched in PAMcluster-C (Fig. 3A, B). In addition, we analyzed the top 10 cancer-related signaling pathways in the three PAM patterns based on the study by Sanchez-Vega et al. [49] (Fig. 3C, Additional file 1: Table S3). The results showed that most of cancer signaling pathways were lowly expressed in PAMcluster-A. And it is worth noting that the cancer signaling pathways of PAMcluster-C are various. Among them, WNT, NOTCH, and MYC were the most enriched, and HIPPO, PI3K and NRF2 expression were the lowest. Further, we used the ssGSEA algorithm to quantify the immune cell infiltration scores of the three PAM patterns to demonstrate the relationship between PAM and immune cells in the TME (Additional file 1: Table S4). Compared with PAMcluster-B and PAMcluster-C, PAMcluster-A had higher levels of CD4 + T cells and CD8 + Tcells, indicating stronger tumor immune function. The dendritic cell (DC) of PAMcluster-C had higher level of NK cells, which may be related to the stronger antigen-presenting ability. Classical immunosuppressive cells including MDSCs, regulatory T cells, follicular helper T cells were significantly elevated in PAMcluster-A, Interestingly, macrophages, B cells, and neutrophils, which are considered to be still controversial for cancer development, were elevated in PAMcluster-C, suggesting significant differences in TME of CRC samples with different polyamine metabolic patterns (Fig. 3D). In line with our expectation, PAMcluster-A was characterized by immune activation and high infiltration of tumor immune cells, while PAMcluster-C was generally immunosuppressed.

### Selecting prognostic differential gene sets and constructing a polyamine metabolism scoring model

**Fig. 4:**
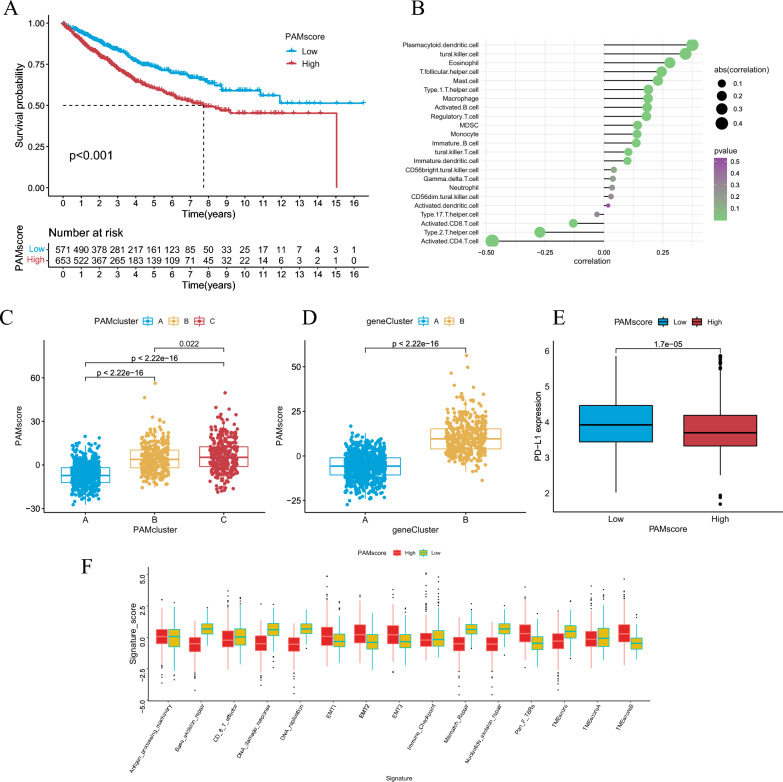
**A** Survival analyses for low and high PAMscore subgroups in the RNA-seq meta cohort. **B** Correlations between PAMscore and TME infiltrating cell abundance in RNA-seq meta cohort using Spearman analysis. The circle size and x-coordinates are used to describe the correlation coefficient. The color of the circle is scaled by P value. **C**, **D** Correlation between PAMscore and geneCluster and PAMscore by the Kruskal–Wallis test. **E** The expression of PD-L1 for different PAMscore subgroup. **F** Boxplot of each TME signature e for high and low PAMscore subgroups in the RNA-seq meta cohort. And the statistical differences among the PAMscore subgroups are tested by the Kruskal–Wallis test. (*P < 0.05; **P < 0.01; ***P < 0.001; ****P < 0.0001)

To quantify the heterogeneity of each CRC sample, we analyzed the DEGs of the three patterns and got the genes that differ between the pairs (Additional file 1: Table S5). Then, we pooled 1742 DEGs according to the changes in transcriptome expression of the three patterns, and selected 328 DEGs with prognostic value according to the above genes through univariate COX regression analysis (Fig. 3E, Additional file 1: Table S6). Based on the 328 DEGs, we could divide them into two clusters by consistency clustering (Additional file 2: Fig. S4A). Therefore, we formed two gene patterns, A and B, and named geneCluster-A and geneCluster-B, respectively (Additional file 1: Table S7). Survival analysis showed that geneCluster-A had a significantly better survival prognosis than geneCluster-B (Fig. 3F). In addition, GO enrichment analysis revealed these 328 DEGs were mainly enriched in DNA replication, cell cycle, and chromosomal genetic features (Additional file 2: Fig. S4B). The KEGG enrichment analysis showed that DEGs were associated with mismatch repair, metabolites and metabolic pathways of various substances (Additional file 2: Fig. S4C). Next, we developed a scoring model by performing PCA analysis based on the 328 prognostic DEGs. Based on this model, each CRC sample was assigned a score termed the polyamine metabolism score (PAMscore). We used “Survminer” package to classify CRC patients into high-PAMscore group and low-PAMscore group (Additional file 1: Table S8), and we used Alluvial diagram to depict the relationship between PAMcluster, geneCluster, PAMscore and survival status (Fig. 3G). The results showed that geneClusterA was mainly derived from PAMclusterA and was the main component of the low PAMscore group, while geneClusterB was mainly derived from PAMclusterC and was the main component of the high-PAMscore group. Survival analysis showed that patients with low-PAMscore had a better survival benefit (Fig. 4A). We conducted a Spearman correlation analysis between PAMscore and various immune cell ssGSEA scores. The results showed that PAMscore was significantly negatively correlated with tumor immune cells CD4 + T cells and CD8 + T cells, and other immune cells were mostly positively correlated with PAMscore (Fig. 4B). It is suggested that PAMscore is closely related to the immunosuppression of CRC. We analyzed the correlation of PAMscore with PAMcluster and geneCluster, and found that, in the PAM pattern, PAMcluster-A with the best prognosis corresponds to the lowest PAMscore, and correspondingly, PAMcluster-A with the worst OS corresponds to the highest PAMscore (Fig. 4C). Likewise, in both A and B gene patterns, the better survival pattern of geneClusterA corresponded to a lower PAMscore (Fig. 4D). We also produced heatmap of the expression levels of the prognostic characteristic gene of both gene patterns (Additional file 2: Fig. S4D). In addition, the expression of PD-L1 in CRC patients between the high and low-PAMscore groups was compared. Compared with the high PAMscore group, the low-PAMscore group had higher PD-L1 expression (Fig. 4E). This suggested that PAMscore may be used to distinguish CRC samples with different responses to immunotherapy. Afterwards, we explored whether there were differences in biological processes in TME between the high and low score groups using previous signature [45] (Fig. 4F). The results showed that multiple tumor suppressor signatures such as Base excision repair, CD8 + T effector, DNA damage response, DNA replication, Mismatch repair, Nucleotide excision repair and TMEscore were significantly increased in the low-PAMscore group, while EMT, Pan-fibroblast TGF-β response signature (Pan-F-TBRS) and TME inhibited signature of TMEscoreB significantly increased in the high-PAMscore group. As expected, the low-PAMscore group was associated with immune-related features, while the high-PAMscore group was associated with immunosuppression and stroma-related features.

### Evaluation of risk characteristics of scores and validation of other cohorts

**Fig. 5:**
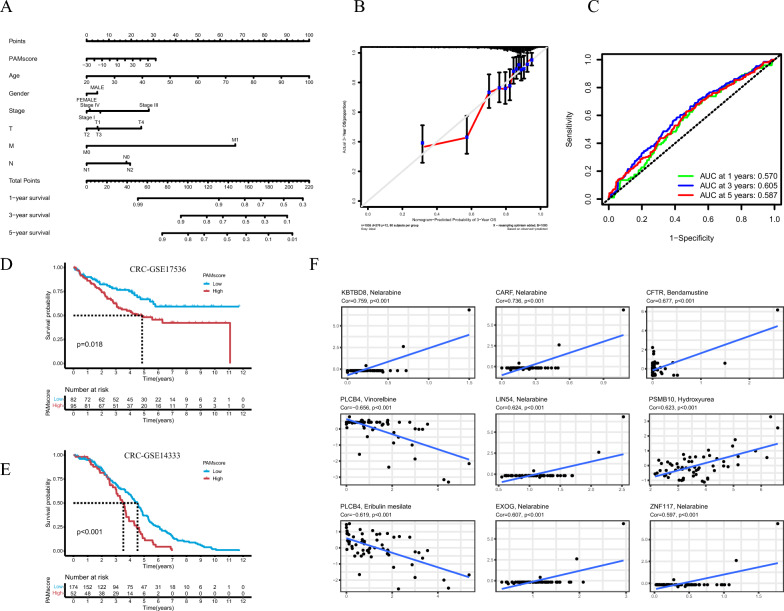
**A** A constructed nomogram for prognostic of a patient with PAMscore. **B** Calibration plot of the nomogram for predicting 3‐year overall survival and cancer-specific survival in meta cohort. **C** ROC curves to predict the sensitivity and specificity of 1-, 3-, and 5-year survival according to the PAMscore. **D**, **E** Validation of Survival analyses for low and high PAMscore subgroups in the other CRC cohorts. **F** Scatter plot of drug sensitivity analysis of genes constructing PAMscore

To better enhance prognostic risk stratification, we constructed a nomogram with PAMscore and clinicopathological features to quantify individual patient risk assessments (Fig. 5A). In addition, calibration analysis was performed to validated the accuracy of the nomogram (Fig. 5B). The results showed that the predicted line of the nomogram is close to actual survival. The AUC of the time-dependent ROC curve at 1-year, 3-year and 5-year overall survival was 0.570, 0.605, 0.587, respectively (Fig. 5C). Next, we validated PAMscore in other CRC cohorts (GSE17536, GSE14333), and the results were as expected, with a significant correlation between low PAMscore and favorable prognosis, confirming that PAMscore is a reliable and independent predictive model. (Fig. 5D, E). We also evaluated PAMscore by univariate and multivariate regression analysis, and the P value was less than 0.05, showing significant differences (Additional file 2: Fig. S4E, F). In addition, we evaluated clinical characteristics using PAMscore based on available CRC clinical cohort information. The results found PAMscore had significant differences in the prediction of 65-year-old age, T3-4, N1, M0 and Stage III group (Additional file 2: Fig. S5). To further analyze the clinical practice of PAMscore, we used the CellMiner database and detected the correlation between PAMscore and drug sensitivity (Fig. 5F, Additional file 1: Table S9). The expression of KBTBD8, CARF, LIN54, EXOG, and ZNF117 had a positive relationship with the sensitivity of Nelarabine (P < 0.001). And the higher the expression of CFTR, the stronger the drug sensitivity of Bendamustine (P < 0.001). The higher the expression of PSMB10, the stronger the drug sensitivity of Hydroxyurea (P < 0.001). PLCB4 had a negative correlation with the sensitivity of Vinorelbine (P < 0.001). And the higher the expression of PLCB4, the weaker the drug sensitivity of Eribulin mesylate (P < 0.001).

### Research of polyamine scoring model with clinical relevance and response to immunotherapy

**Fig. 6:**
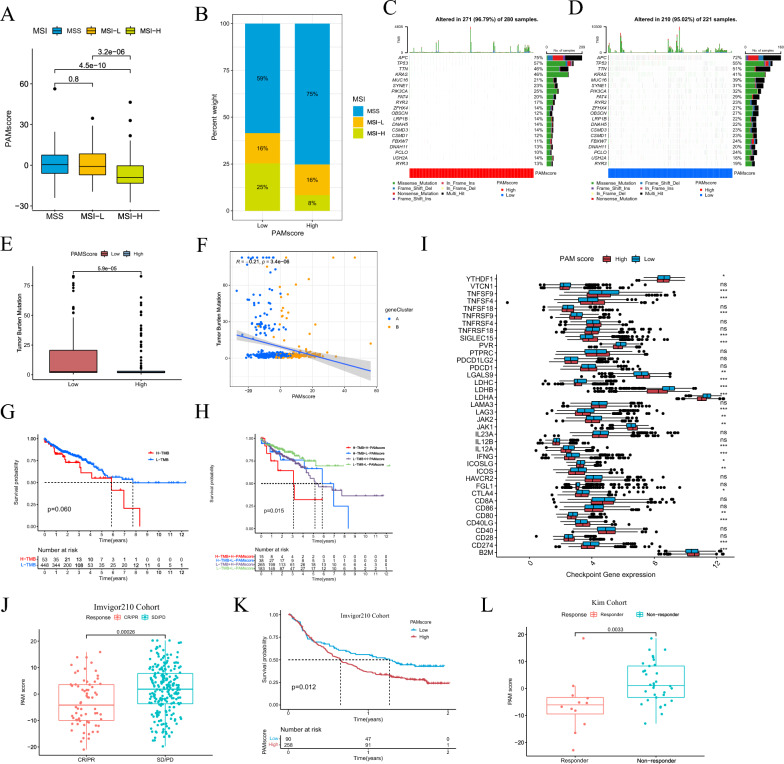
**A** Relationship between MSI and PAMscore in CRC patients **B** The proportion of patients with microsatellite instability in high and low subgroups of PAMscore **C**, **D** Waterfall chart showing gene mutation in high and low PAMscore groups. **E**, **F** Correlation between tumor mutational burden (TMB) and PAMscore. **G**, **H** TMB and TMB combined with PAMscore to predict the survival prognosis of CRC patients with TCGA respectively. **I** Boxplots of differences in gene expression of major immune checkpoints between high and low PAMscore subgroups. **J**, **L** PAMscore prediction of response to Imvigor210 cohort in bladder cancer and Kim cohort in gastric cancer. **K** Survival analyses for low and high PAMscore subgroups in Imvigor210 cohort of bladder cancer

Our analysis revealed differences in survival prognosis between the high- and low-PAMscore subgroups. Based on this, we further explore the relevant mechanisms and internal connections of these results. MSI and TMB are of significant value in the prognostic judgment, medication evaluation and immunotherapy prediction of CRC. Therefore, we performed a correlation analysis of MSI status and TMB of CRC samples from TCGA. From the PAMscore, the microsatellite stability (MSS) and MSI-L status had higher scores, while MSI-H corresponded to lower scores (Fig. 6A). The proportions of MSS and MSI-H were also different in the high- and low-PAMscore subgroup (Fig. 6B). In the high-PAMscore subgroup, MSS accounted for 75% of patients compared with 8% of MSI-H patients; in the low-PAMscore subgroup, MSS patients accounted for 59% and MSI-H patients accounted for 25%. Subsequently, we further performed a significantly mutated gene (SMG) analysis of the CRC samples in the high- and low-PAMscore subgroup (Fig. 6C, D). The SMG mutation map indicated that the somatic mutation signature was overall higher in the low-PAMscore subgroup than in the high-PAMscore subgroup, among which, MYC16 (39% vs 21%), SYNE1 (37% vs 23%), PIK3CA (32% vs 25%) %) and FAT4 (29% vs 20%) had higher mutation rates in the low-PAMscore subgroup. These results suggested that PAMscore was closely associated with genomic variation in CRC and reflected its clinical relevance in predicting potential responses to immunotherapy.

Based on the association between somatic mutations in tumor genomes and response to immunotherapy, we explored TMB distribution patterns in the high- and low- PAMscore groups. As expected, the results showed that the low-PAMscore subgroup had an extremely high frequency of tumor mutations compared to the high-PAMscore subgroup (Fig. 6E). Furthermore, we combined geneCluster to analyze the degree of correlation between TMB and PAMscore, and found that TMB and PAMscore showed a strong negative correlation (Fig. 6F). Based on the predictive ability of TMB for CRC patients, we further explored whether PAMscore could enhance its survival predictive ability (Fig. 6G, H). The results showed that the low TMB plus low-PAMscore groups had the best prognosis, while the high TMB plus high-PAMscore groups had the worst prognosis, and the remaining two groups were in the middle. This fully confirmed the favorable predictive ability of PAMscore combined with TMB.

From the characteristic results of MSI and TMB in PAMscore, we believed that the scoring model of PAM in tumors played a crucial role in mediating immune responses and predicting immunotherapy. To investigate this, we first performed a correlation analysis of the expression levels of immune checkpoint genes in high- and low-PAMscore subgroups (Fig. 6I). Most of the immune checkpoint genes were significantly different between the two groups, and important immune checkpoints such as CD274, CD80, ICOS, FNG, IL-12A, JAK1, JAK2, LAG3, PVR were highly expressed in the low-PAMscore subgroup. These results correlate strongly correlate with immunotherapy response. To further investigate, we used PAMscore to predict patient response to Immune checkpoint inhibitor (ICI) treatment in two independent immunotherapy cohorts. In the Imvigor210 cohort of bladder cancer, all patients treated with atezolizumab, we analyzed the relationship between PAMscore and immunotherapy response and the prediction of survival benefit in this cohort. It was clearly found that there was a significant difference in PAMscore between CR/PR and SD/PD patients. Consistent with our conjecture, CR/PR patients corresponded to a lower PAMscore, while SD/PD patients corresponded to a higher PAMscore (Fig. 6J). We also used PAMscore to perform a survival prognostic analysis of the Imvigor210 cohort, and the results also showed that the low-PAMscore subgroup had a better survival benefit (Fig. 6K). Furthermore, we analyzed the relationship between PAMscore and immunotherapy response in the gastric cancer Kim cohort treated with Pembrolizumab, and the results showed that the PAMscore of responders was significantly lower than that of non-responders (Fig. 6L). The above results demonstrated that patients with low PAMscore could acquire more advantage and greater benefit from ICI treatment, and the PAMscore could also improve the prediction of response to anti-PD-L1 or anti-PD1 immunotherapy.

### Screening and identification of characteristic genes

**Fig. 7:**
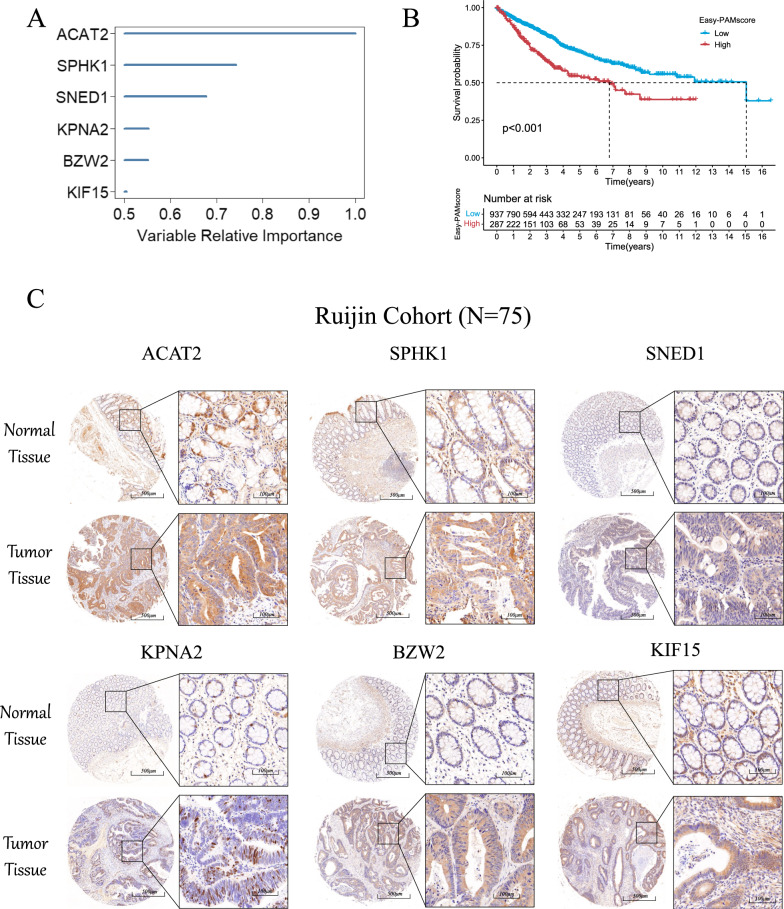
**A** Hub genes in the prognostic genes of Easy-PAMscore obtained by random forest (Relative importance threshold < 0.4). **B** Survival prognostic analysis of meta cohorts using hub genes. **C** Validation of the characteristic genes (ACAT2, SPHK1, SNED1, KPNA2, BZW2, KIF15) in CRC clinical samples by immunohistochemistry

We screened the 328 genes used to construct the PAM scoring model through the random forest method to obtain representative characteristic genes. We used the "randomForestSRC" package for feature selection. We determined the genes with relative importance threshold < 0.4 as the final features and ranked them according to their importance. Finally, six feature genes of ACAT2, SPHK1, SNED1, KPNA2, BZW2 and KIF15 were identified. (Fig. 7A, Additional file 1: Table S10). We used these six genes to construct Easy-PAMscore using the same method and again performed a survival prognostic analysis of high and low PAMscore for patients in the CRC cohort. The results still showed that the low-PAMscore subgroup was able to achieve better survival than the high-PAMscore subgroup (Fig. 7B). To validate the expression of the above genes in normal and tumor tissues of CRC, we first analyzed the RNA expression levels of six genes in TCGA (Additional file 2: Fig. S6A). We discovered the expression of all genes except SNED1 were basically higher in tumors than in normal tissues. More specifically, 75 pairs of CRC tissues from the Shanghai Minimally Invasive Surgery Center of Ruijin Hospital (Shanghai, China) were analyzed by IHC staining. We performed immunohistochemical analysis of ACAT2, SPHK1, SNED1, KPNA2, BZW2 and KIF15 in cancer and normal tissues and statistically analyzed the staining intensity (Additional file 2: Fig. S6B–G). The results indicated that six characteristic genes of the signature were generally overexpressed in the CRC tissues compared to the normal tissues (Fig. 7C).

### Single-cell analysis reveals the characteristic of polyamine metabolism in the TME

**Fig. 8:**
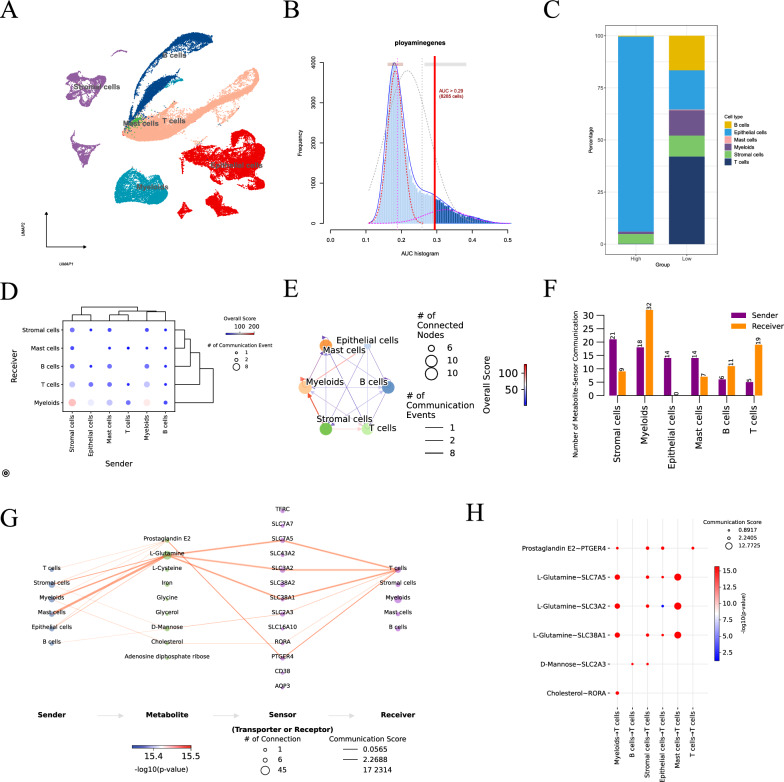
**A** UMAP plot for identification of 6 types of cells in GSE132465. **B** AUC histogram for polyamine genes divided cells into high and low groups. **C** Distribution of 6 cell types in high and low groups. **D** Bubble diagram demonstrated cell–cell communication event. **E** The interconnections and nodes of cell–cell communication. **F** Bar graph of number of metabolite-sender communication in 6 cell types. **G** The network of metabolites and molecules that communicate between cells. **H** Communication score of the contact for metabolites and molecules in cell–cell communication

Single-cell analysis was performed to reveal and verify the crucial effects of PAM in the TME. To depict the landscape and feature, single-cell transcriptomes from GSE132465 were analyzed with subcluster analysis and visualized using UMAP approach. As shown in the UMAP plot, we manually annotated these clusters as the following 6 cell types: B cells, epithelial cells, mast cells, Myeloid cells, stromal cells and T cells (Fig. 8A). The expression level of PAM genes based on PAM score was delivered into an AUC scoring algorithm, and eventually over 60,000 cells were assigned into high/ low-AUCscore group (Fig. 8B). We further measured the percentage composition in the high/low-AUCscore group and extracted the top 6 markers of each immune cells cluster. In the high-AUCscore group, epithelial cells were in the majority, and immune cells such as T cells and B cells, which were considered to have favorable prognostic characteristics, dominated in the low-AUCscore group (Fig. 8C, Additional file 2: Fig. S7A). We also demonstrated the markers of 6 immune cell clusters in the high/low-AUCscore group (Additional file 2: Fig. S7B). To further assess the interaction of the immune cells in the CRC patients under the background of PAM, we investigated the cell–cell communication conditions. To this end, we evaluated the putative crosstalk of 6 immune cells with the R package “CellChat”. We found that the interaction between several cell clusters was frequent, and both Myeloid cells and T cells received signals from other cell clusters, especially stromal cells as the “sender” had strong communication with Myeloid cells as the “receiver” (Fig. 8D, E). We conducted the correlation analysis to elucidate the communication between six cell clusters and metabolites (Fig. 8F). In the framework of immune cell clusters, the role and association of metabolites might be the crucial way for cell–cell communication. For this purpose, we analyzed the complex network of intercellular metabolites and related molecules (Fig. 8G, H, Additional file 2: Table S11). In this network, the core corresponding metabolites include Prostaglandin E2, l-Glutamine, d-Mannose and Cholesterol with the 6 cell types starting. The pivotal “Transporter” contained SCL7A5, SCL3A2, SCL38A1, SCL2A3, RORA, and PTGER4, and the final “Receiver” was directed to T cells. Myeloid cells to T cells and Mast cells to T cells have a more prominent status in the overall communication network, and the most advantageous communication score focused on l-Glutamine-SLC7A5, l-Glutamine-SCL3A2 and l-Glutamine-SCL38A1. These results suggested underlying mechanisms of between PAM and TME for the cell–cell communication status and the related metabolites functions.

## Discussion

Plentiful evidence indicated that metabolic reprogramming deeply influences tumorigenesis, progression, biology pathways, drug sensitivity, and immunotherapy response in cancer [50–52]. Polyamines were quite abundant in the internal and external environment, especially in the digestive tract, where they were absorbed and transported to the cells throughout the body [53]. It was reasonable to speculate that polyamines may be involved in the tumorigenesis and progression of digestive diseases. Recently, multiple studies have pointed to the involvement of PAM in adaptive immunity, TME transformation and cancer-related inflammation [20]. These phenotypes were not only associated with the prognosis of cancer patients, but provided theoretical support for targeted therapy or immunotherapy. Therefore, the specific relationship between PAM and cancer was in urgent need of further analysis and elucidation. It was necessary to illustrate systematic and holistic analysis in specific cancer types to obtain novel treatment strategies.

In this study, we first analyzed the characteristics of PAM in CRC and found that the vast majority of PAM genes were significantly overexpressed in tumors compared with normal tissues, and the corresponding CNV detection also showed obvious changes. Further, we identified three different PAM patterns by utilizing unsupervised consistent clustering and divided patients into PAMcluster-A, B and C. Based on the survival analysis of the patterns, we conducted a corresponding analysis of related pathways and immune infiltration. The results showed that the characteristics of PAMcluster-A which had the best survival probability and related immune infiltration of CD4 + and CD8 + T cells, and weak expression of most cancer pathways within it. On the other hand, PAMcluster-C, which had the worst survival probability, had a variety of immunosuppressive cell infiltration, including MDSC, Tregs, mast cells, and significantly activated cancer-related pathways such as WNT, NOTCH, and MYC pathways, which was consistent with previous studies [54–56]. PAMcluster-B, which had a moderate survival probability, was significantly higher expressed in cancer-related pathway including NRF-2, RTK/RAS, TGF-β compared A and C. Activation of these signaling pathways and increased levels of immunosuppressive cell infiltration both predicted poor prognosis and rapid progression of cancer, confirming an essential association and possible mechanisms of polyamine metabolism in cancer signal pathway and TME remodeling.

Based on these results, we identified DEGs from three PAM patterns and found that DEGs were associated with a variety of substances metabolism and genomic modifications. That suggested these DEGs were highly correlated between PAM and CRC. Accordingly, we further constructed the "PAMscore" model to validate CRC samples to more accurately predict patient prognosis and improve individualized treatment strategies. The verification results showed the high PAMscore subgroup had a worse prognosis, while the low PAMscore subgroup had a better prognosis, which was consistent with our expectation that higher intensity of PAM is associated with a worse survival outcome. In the signature of TME, the high PAMscore subgroup was closely related to EMT, PAN-F-TBRS, immunosuppression score TMBscoreB, and Antigen processing Machinery. Base Excision repair, CD8 + T cell effector, DNA damage response and DNA replication closely related the low PAMscore subgroup [45]. These results indicated that PAMscore could be used to describe CRC patients’ characteristics. Further analyses of other CRC cohorts highlighted PAMscore as an effective prognostic model for CRC. In addition, considering the close relationship between PAM and TME, the association of related genes with small-molecule drugs were also estimated, which included Nelarabine, Bendamustine, Vinorebline, Hydroxyurea and Eribulin mesylate.

Other than elucidating the predictive ability of PAMscore, we explored in detail the relationship between PAMscore and clinical immunotherapy for CRC. MSI status was an important predictor of prognosis and immunotherapy response in CRC patients [8]. Consistently, the high-PAMscore subgroup possessed a significantly higher proportion of MSS patients and lower TMB than that in the low-PAMscore subgroup, which might account for the unfavorable response of high-PAMscore patients to ICI treatment in two cohorts. Besides, we observed higher mutation rates of typical oncogenes, such as MUC16, SYNE1, PIK3CA and FAT4, in the low-PAMscore subgroup in mutation analysis, which may result in higher TMB. In addition, immune checkpoint gene expression was generally higher in the low PAMscore subgroup, which led us to propose that synergistic blockage of key genes in PAM related pathways and immune checkpoint might be a promising strategy to overcome the resistance of ICI [57, 58]. To this end, we selected the core genes based on the DEGs of PAMscore construction and verified them histologically. ACAT2 and SPHK1 were related to lipid metabolism and some tumor-promoting pathways [59, 60], SNED1 might be related to cell matrix adhesion [61]. KPNA2, BZW2 and KIF15 were involved in protein synthesis and transport and were suggested to be related to tumor progression [62–64]. These key genes would have upstream and downstream relationship with PAM and participated in the related mechanisms of PAM regulating cancer. The PAMscore model established a classification and decision-making framework for CRC patients based on a scoring system. It validated the significant impact and potential involvement of polyamine metabolism in CRC prognosis, clinical features, TME, and immunotherapy. Notably, PAMscore highlights its clinical prognostic capacity from the perspective of polyamine metabolism, which may inform the identification of potential therapeutic targets or the enhancement of immunotherapeutic efficacy in CRC. Therefore, PAMscore is a valuable addition to future comprehensive clinical prediction models.scRNA-seq technology has enabled discovery of distinct cell subsets, characterization of cell interactions, and identification of key factors in tumor heterogeneity [65]. However, the microscopic intercellular crosstalk in CRC was unknown, and a detailed description of the TME landscape in the context of PAM was absent. In the single-cell cluster and AUC scoring analysis, we found that epithelial cells accounted for the vast majority in the high-AUCsocre group. It was closely related to polyamines that maintain the active state of tumor cells and promote tumor cell proliferation. Myeloid cells and stromal cells which thought to be immunosuppressive cells in TME, had the most active communication with cancer cells in our analysis. According to the result of cell–cell communication, we constructed a communication network between cells and metabolites. Previous studies have confirmed that glutamine is involved in tumorigenesis and progression and affects the metabolism of tumor cells [66]. The core metabolite in the network we constructed was l-glutamine exactly. SLC7A5, SLC3A2 and SLC38A1, critical members of the solute carrier family, were involved in the transport of amino acid metabolites as "Transporter". They were also used for T cell immunity and remodeling the immunosuppressive TME to promote tumor growth. Imbalanced polyamine levels affected the function of TME through cell and metabolite communication, and then participated in a variety of cellular processes of tumor cells. Moreover, multiple cancer types of immunotherapy cohorts for PAMscore still need to be explored, and a prospective CRC cohort should be used to validate the prediction model. What’ s more, the regulatory mechanisms of metabolites and key targets of PAM, such as ODC1 and proteasome family genes, still need to be further studied. Future explorations are necessary to answer these questions.

## Conclusion

Taken together, we described the characteristics of PAM genes in CRC and evaluated the PAM patterns in CRC samples based on PAM genes. We constructed a PAMscore prognostic model to systematically and comprehensively describe CRC prognosis, TME, and immunotherapy. The immune landscape of polyamine metabolism was also been demonstrated in single-cell level. These results provided theoretical basis for further searching for key metabolite therapeutic targets and understanding the regulation of tumor immunity. More broadly, by highlighting the critical role and prognostic value of polyamine metabolism, our study may pave the way for the development of novel therapeutic strategies against CRC.

## Supplementary Information

**Additional file 1: Table S1.** Summary of Polyamine Meatabolismrelated genes. **Table S2.** Samples clustering in CRC RNA-seq meta cohorts. **Table S3.** List of 10 major signaling pathway genes associated with cancer. **Table S4.** ssGSEA results for immune cells of meta cohort. **Table S5.** Differental PAM related genes for PAMcluster. **Table S6.** The gene of construction PAMscore. **Table S7.** Samples clustering in CRC RNA-seq meta cohorts. **Table S8.** The gene of construction PAMscore. PAMscore subgroup analysis by constructing a PAM scoring model. **Table S9.** Drug susceptibility results corresponding to PAMscore model genes. **Table S10.** Importance results of random forest screening. **Table S11.** Communication of immune cell based on PAMSscore model genes.

**Additional file 2: Figure S1.**Boxplot of the expression of PAM genes in tumor and normal sample on the TCGA cohort.Heatmap of significant different PAM genes in tumor and normal sample..GO enrichment analysis for PAM genes.KEGG enrichment analysis for PAM genes.Forest plot of prognostic gene with Univariate cox regression analysis.Protein–protein interactionfor PAM-related genes.Venn diagram showing PAM genes after intersection of datasets. **Figure S2.** Survival prognostic analysis of each PAM gene with high and low expression using Kaplan–Meier analysis. **Figure S3.**Consensus clustering of 55 PAM genes matrix for k = 3 of 1224 patients in the TCGA cohort combine the GEO cohort.Determine the relevant CDF curve and Tracking plot of Consensus clustering. **Figure S4.**Consensus clustering of 328 PAM prognostic genes in the meta dataset.GO enrichment analysis and KEGG enrichment analysis of 328 PAM prognostic genes.Expression heatmap of 328 PAM prognostic genes in geneCluster A and B subgroups.Forest plots for univariate and multivariate cox analysis of PAMscore. **Figure S5.** Survival analysis of high and low score subgroups of PAMscore for different genders, different T, N, M stages and AJCC stages in CRC patients. **Figure S6.**RNA expression of ACAT2, SPHK1, SNED1, KPNA2, BZW2 and KIF15 in tumor and normal tissues in TCGA dataset.IHC cell staining intensity in normal and tumor tissues of the CRC cohort. **Figure S7.**Expression levels of marker genes in the 6 cell types.Expression levels of marker genes in high and low cell groups.

## Data Availability

The datasets used and/or analyzed during the current study are available from the corresponding author upon reasonable request.

## Abbreviations

CRC: Colorectal cancer
PD1: Programmed cell death 1
PD-L1: Programmed cell death-ligand 1
CTLA4: Cytotoxic T-lymphocyte antigen 4
MMR: Mismatch repair tumors
TME: Tumor microenvironment
CEA: Carcinoembryonic antigen
PAM: Polyamine metabolism
scRNA-seq: Single-cell RNA sequencing
TCGA: The Cancer Genome Atlas
GEO: Gene Expression Omnibus
OS: Overall survival
FPKM: Fragments per kilobase of transcript per million mapped reads
TPM: Transcripts per million
GSVA: Gene set variation analysis
GO: Gene ontology
ssGSEA: Single-sample gene set enrichment analysis
DEG: Differential gene
PCA: Principal component analysis
IHC: Immunohistochemistry
ROC: Receiver operating characteristic
PPI: Protein–protein interaction
CNV: Copy number variation
EMT: Epithelial–mesenchymal transition
Pan -F-TBRS: Pan-fibroblast TGF-β response signature
AUC: Area under the curve
MSI: Microsatellite instability
TMB: Tumor mutational burden
MSS: Microsatellite stability
SMG: Significantly mutated gene
ICI: Immune checkpoint inhibitor
CR: Complete remission
PR: Partial remission
SD: Stable disease
PD: Progressive disease
UMAP: Uniform Manifold Approximation and Projection
