# Supplementary figures and images for: Polyamine metabolism patterns characterized tumor microenvironment, prognosis, and response to immunotherapy in colorectal cancer
Source: Cancer Cell Int. 2023 May 18;23:96. doi: 10.1186/s12935-023-02892-z (PMC10197465; doi:10.1186/s12935-023-02892-z)

A

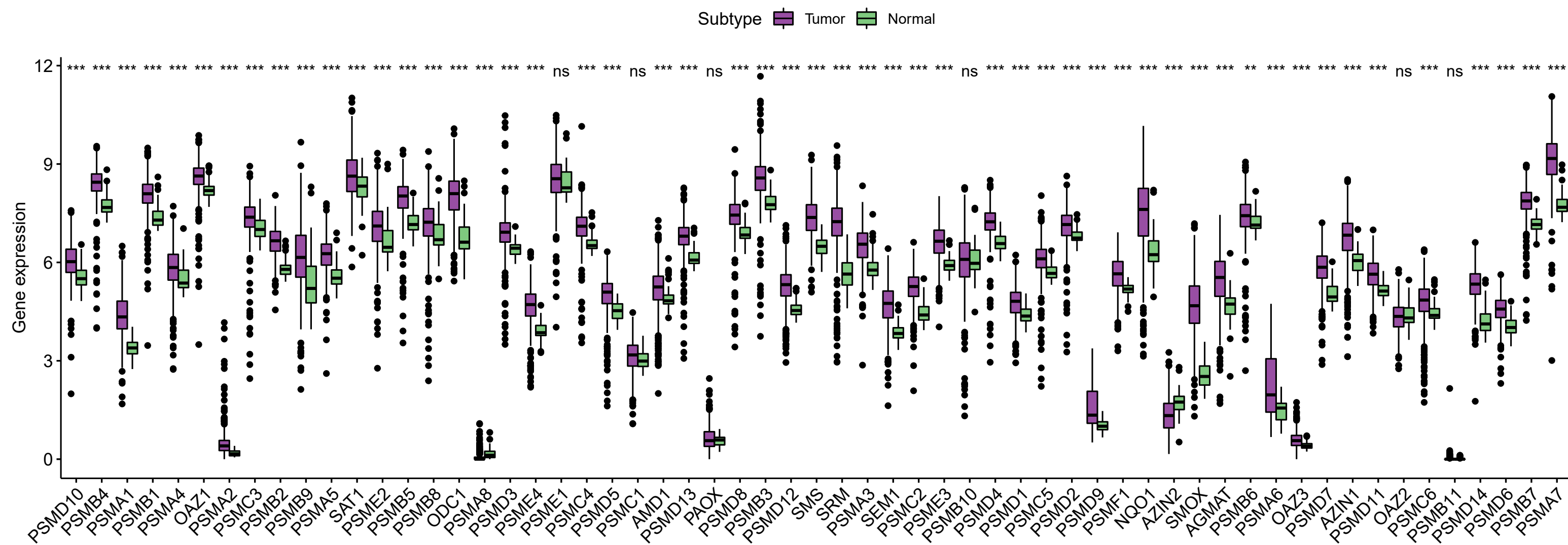

C

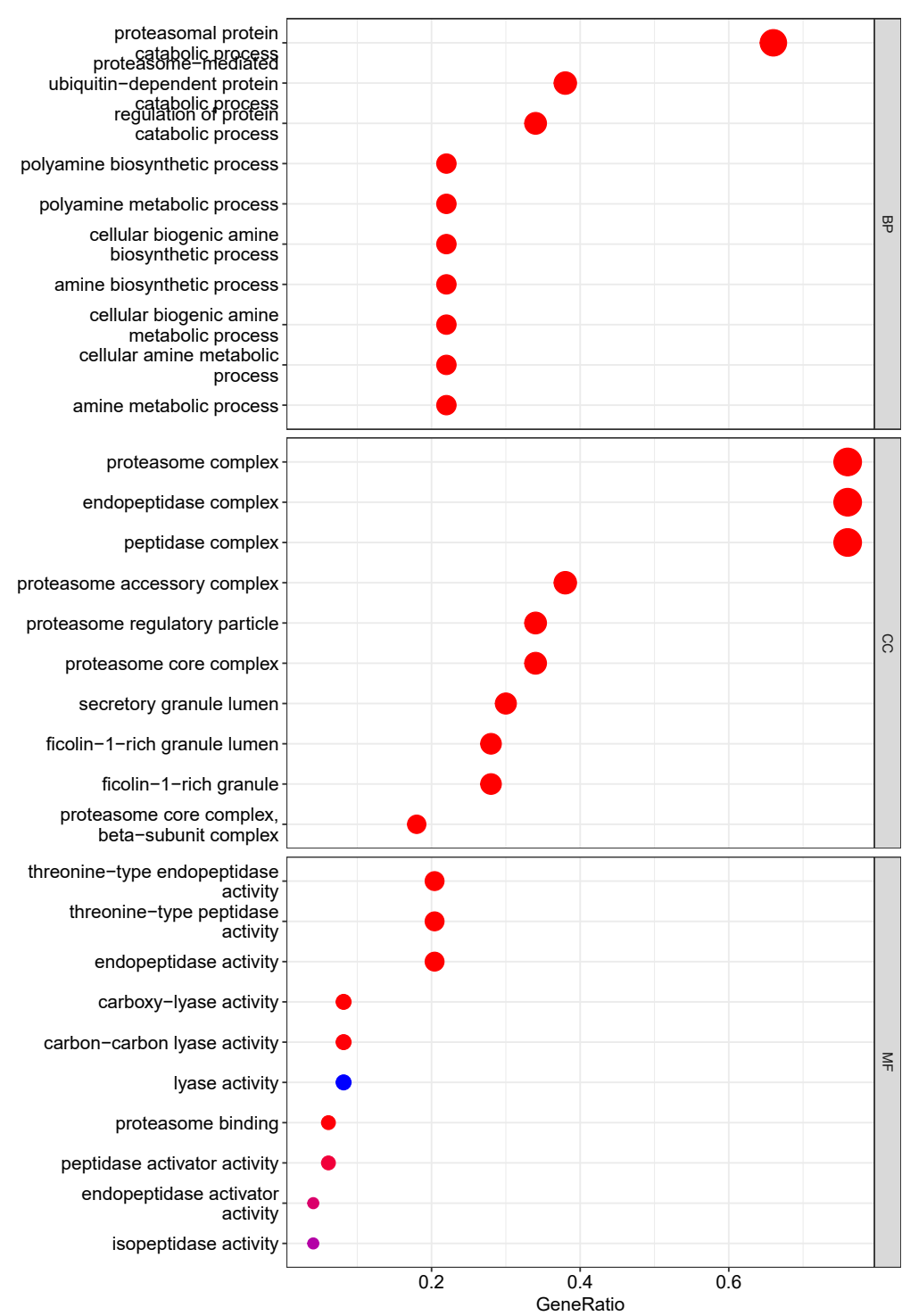

D

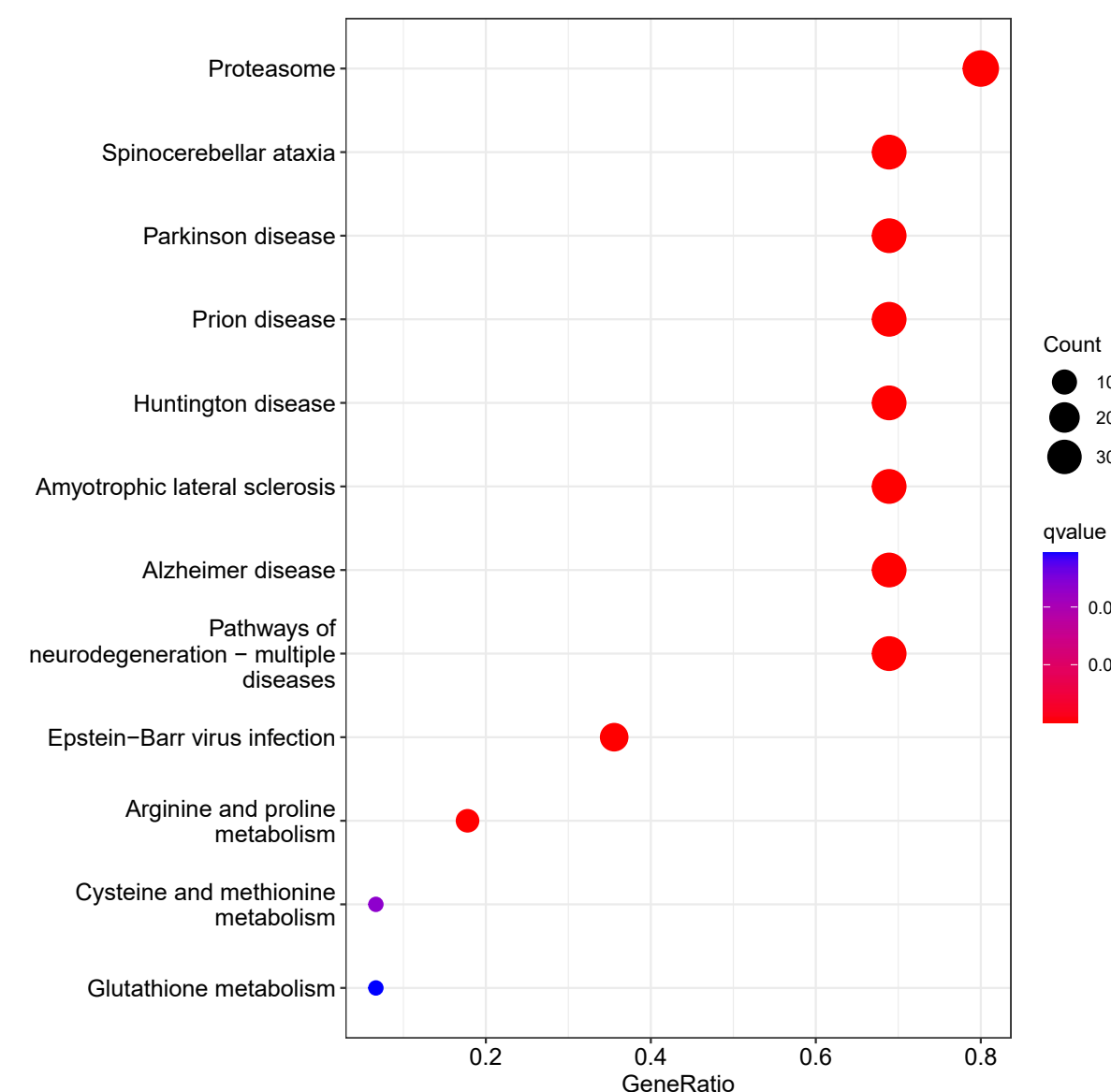

B

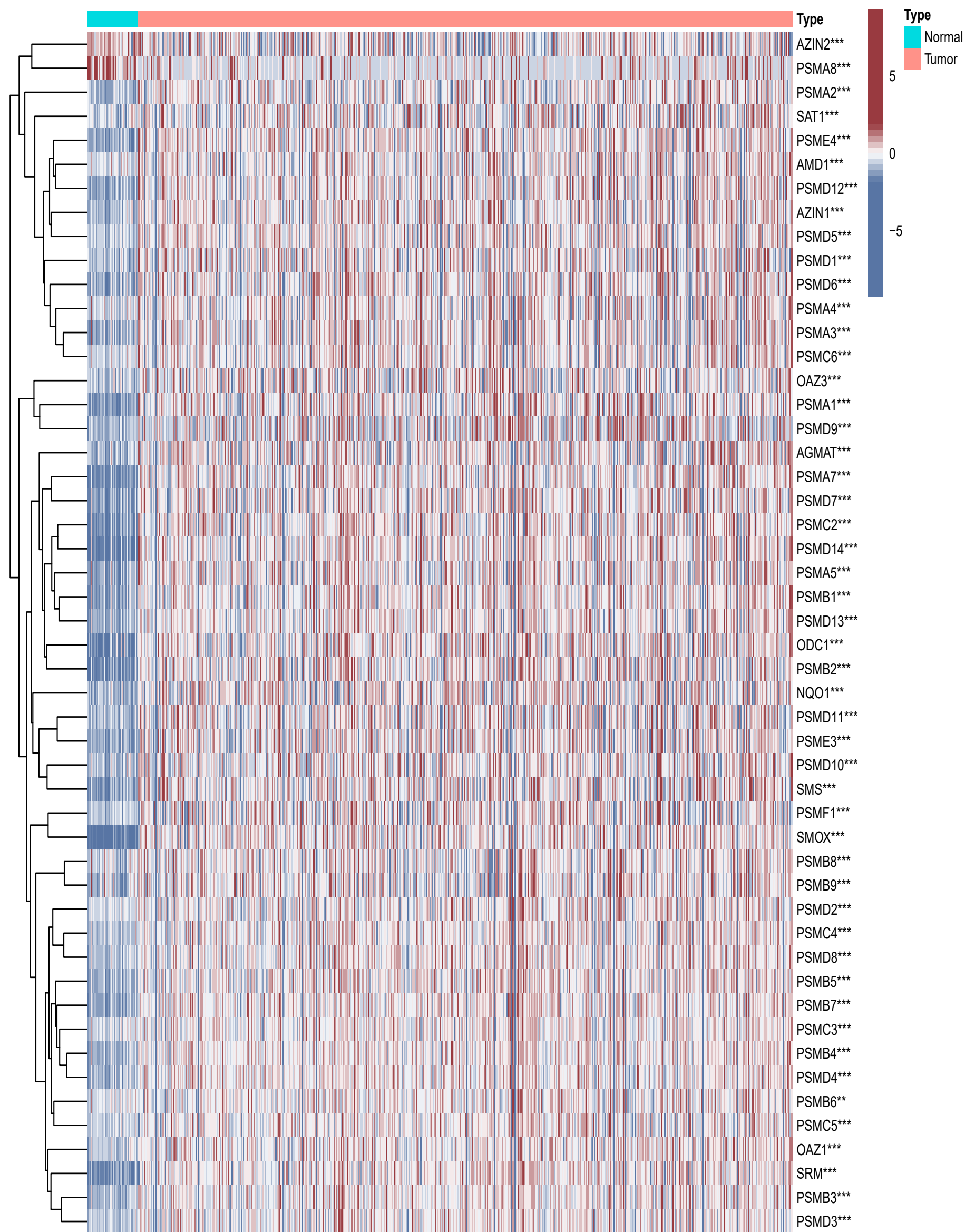

E

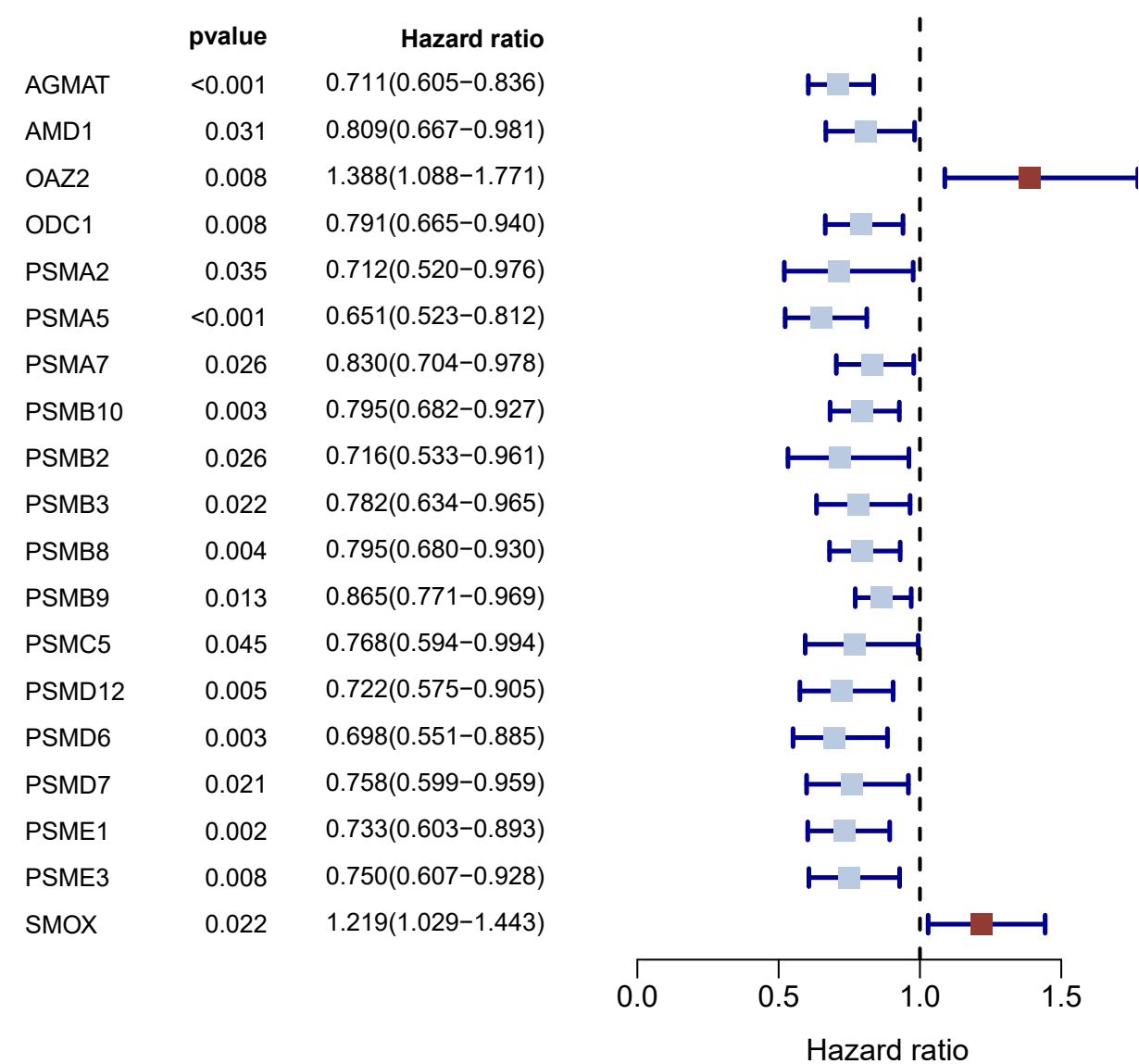

F

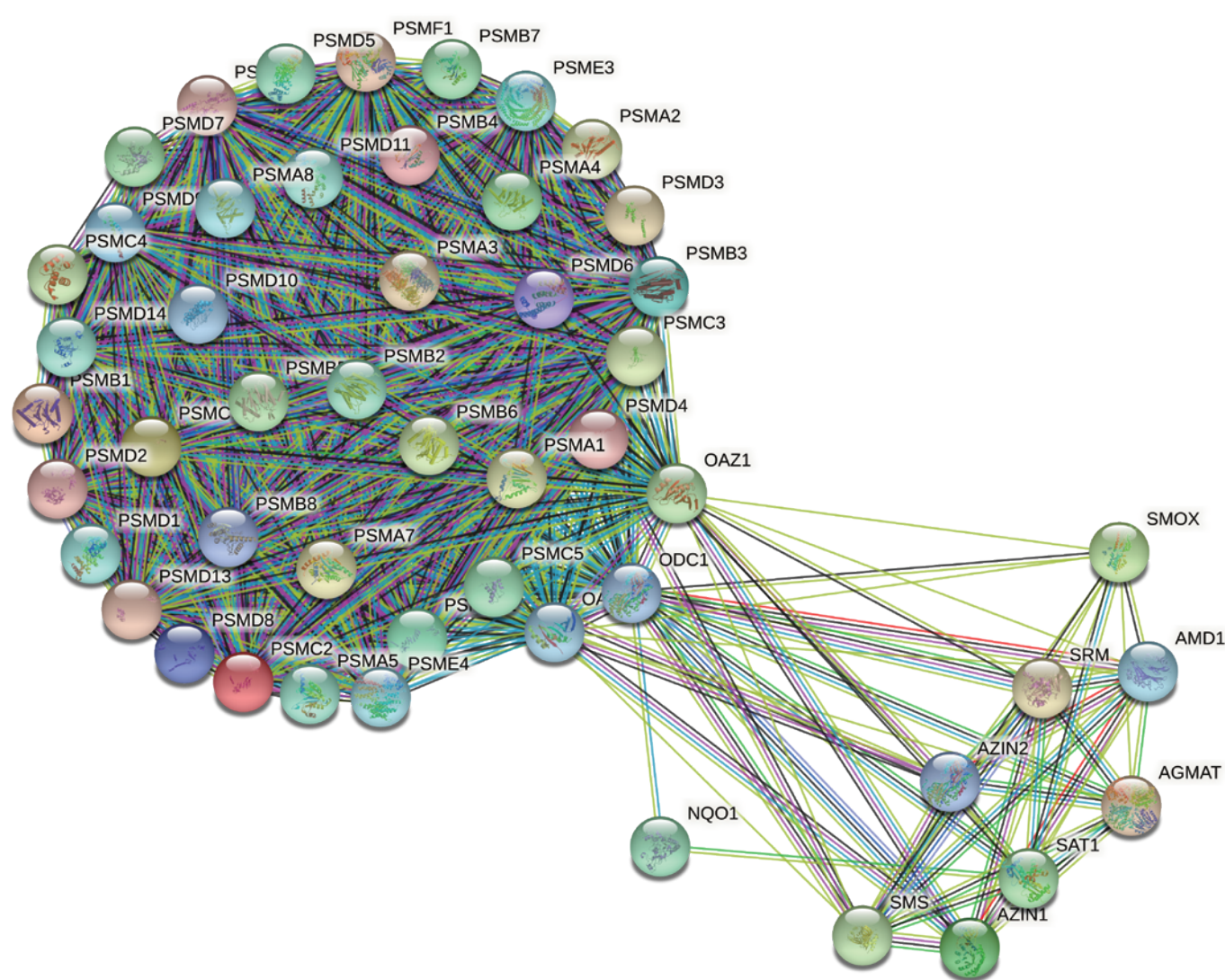

G

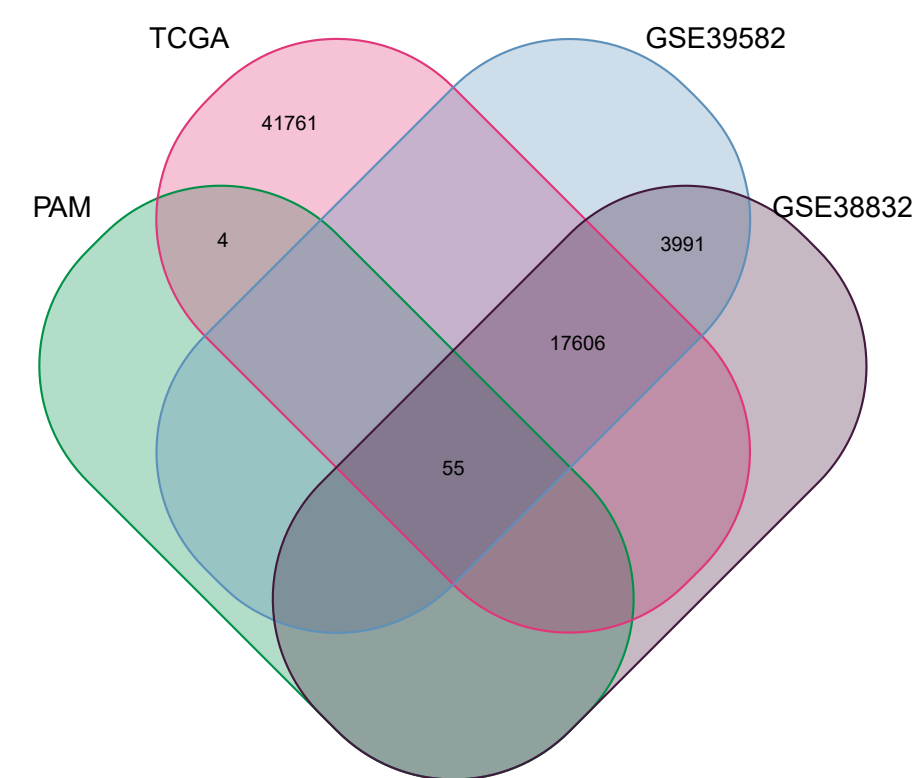

Supplement: Supplementary file 2 — Additional file 2: Figure S1.Boxplot of the expression of PAM genes in tumor and normal sample on the TCGA cohort.Heatmap of significant different PAM genes in tumor and normal sample..GO enrichment analysis for PAM genes.KEGG enrichment analysis for PAM genes.Forest plot of prognostic gene with Univariate cox regression analysis.Protein–protein interactionfor PAM-related genes.Venn diagram showing PAM genes after intersection of datasets. Figure S2. Survival prognostic analysis of each PAM gene with high and low expression using Kaplan–Meier analysis. Figure S3.Consensus clustering of 55 PAM genes matrix for k = 3 of 1224 patients in the TCGA cohort combine the GEO cohort.Determine the relevant CDF curve and Tracking plot of Consensus clustering. Figure S4.Consensus clustering of 328 PAM prognostic genes in the meta dataset.GO enrichment analysis and KEGG enrichment analysis of 328 PAM prognostic genes.Expression heatmap of 328 PAM prognostic genes in geneCluster A and B subgroups.Forest plots for univariate and multivariate cox analysis of PAMscore. Figure S5. Survival analysis of high and low score subgroups of PAMscore for different genders, different T, N, M stages and AJCC stages in CRC patients. Figure S6.RNA expression of ACAT2, SPHK1, SNED1, KPNA2, BZW2 and KIF15 in tumor and normal tissues in TCGA dataset.IHC cell staining intensity in normal and tumor tissues of the CRC cohort. Figure S7.Expression levels of marker genes in the 6 cell types.Expression levels of marker genes in high and low cell groups. [file 12935_2023_2892_MOESM2_ESM.zip › Supplementary Material 2/S1.pdf]

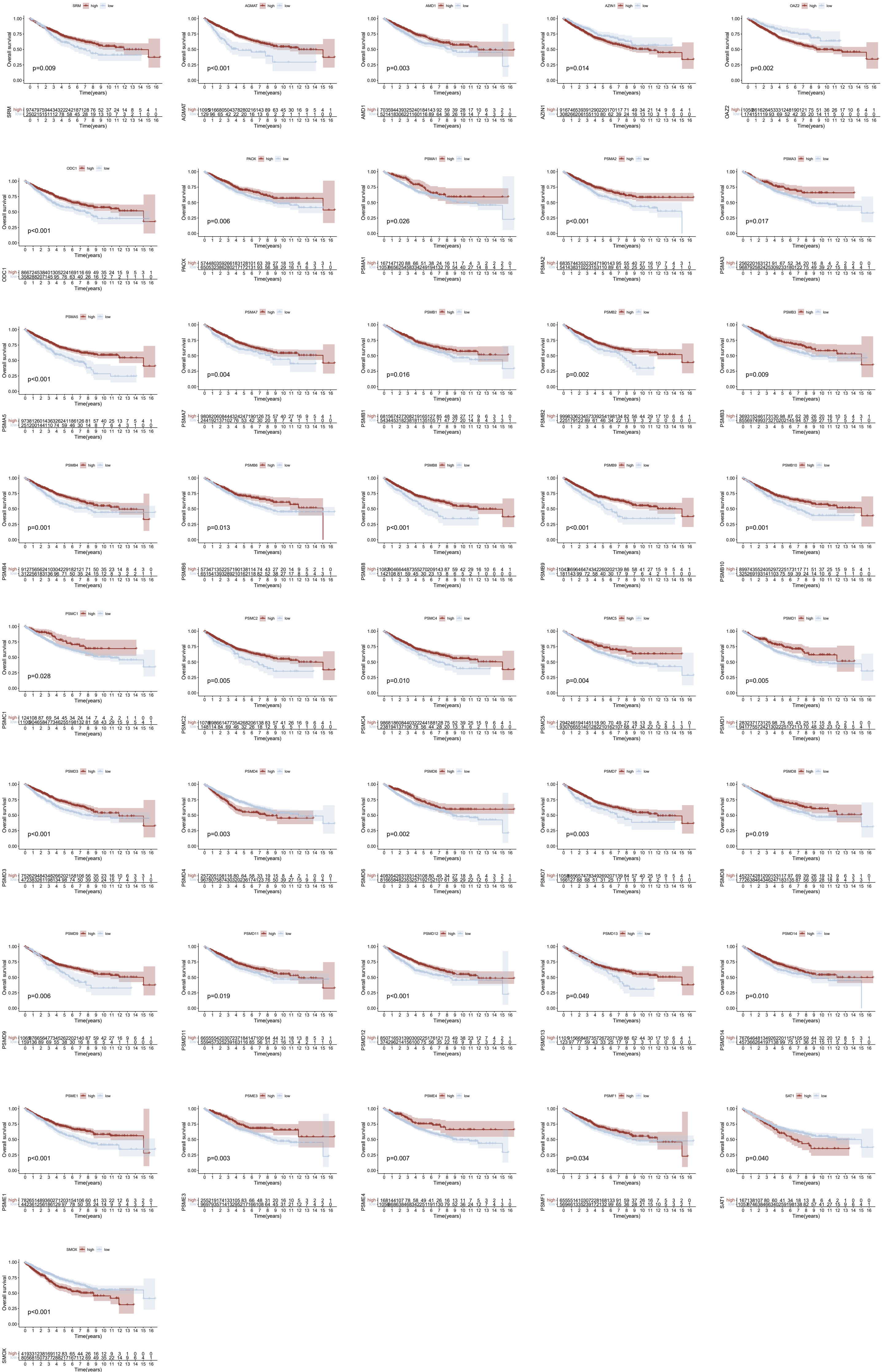

Supplement: Supplementary file 2 — Additional file 2: Figure S1.Boxplot of the expression of PAM genes in tumor and normal sample on the TCGA cohort.Heatmap of significant different PAM genes in tumor and normal sample..GO enrichment analysis for PAM genes.KEGG enrichment analysis for PAM genes.Forest plot of prognostic gene with Univariate cox regression analysis.Protein–protein interactionfor PAM-related genes.Venn diagram showing PAM genes after intersection of datasets. Figure S2. Survival prognostic analysis of each PAM gene with high and low expression using Kaplan–Meier analysis. Figure S3.Consensus clustering of 55 PAM genes matrix for k = 3 of 1224 patients in the TCGA cohort combine the GEO cohort.Determine the relevant CDF curve and Tracking plot of Consensus clustering. Figure S4.Consensus clustering of 328 PAM prognostic genes in the meta dataset.GO enrichment analysis and KEGG enrichment analysis of 328 PAM prognostic genes.Expression heatmap of 328 PAM prognostic genes in geneCluster A and B subgroups.Forest plots for univariate and multivariate cox analysis of PAMscore. Figure S5. Survival analysis of high and low score subgroups of PAMscore for different genders, different T, N, M stages and AJCC stages in CRC patients. Figure S6.RNA expression of ACAT2, SPHK1, SNED1, KPNA2, BZW2 and KIF15 in tumor and normal tissues in TCGA dataset.IHC cell staining intensity in normal and tumor tissues of the CRC cohort. Figure S7.Expression levels of marker genes in the 6 cell types.Expression levels of marker genes in high and low cell groups. [file 12935_2023_2892_MOESM2_ESM.zip › Supplementary Material 2/S2.pdf]

A

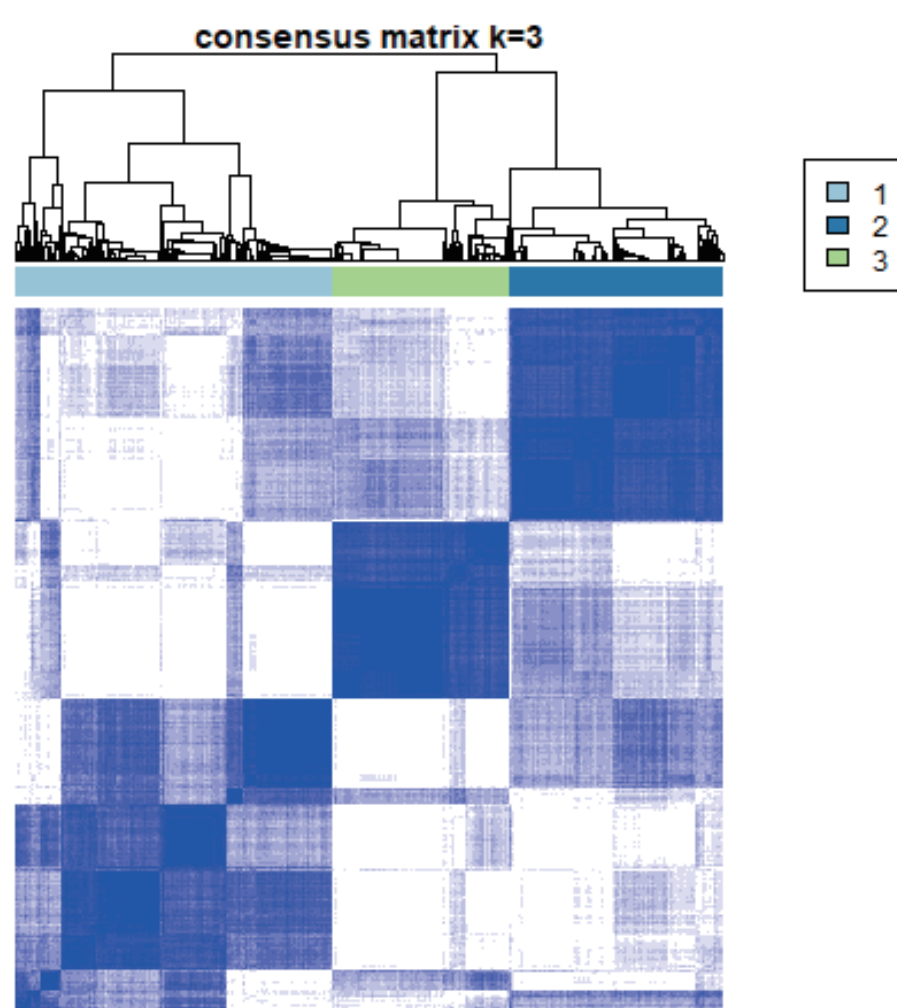

B

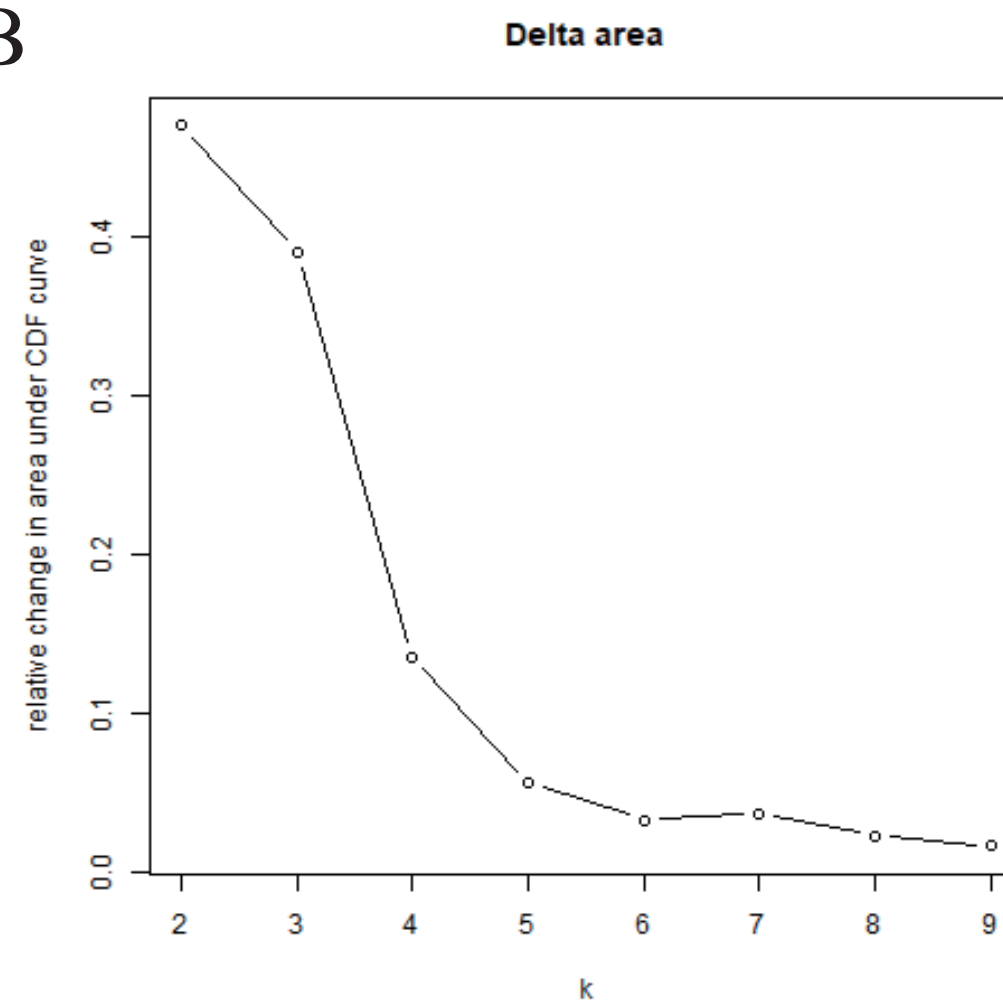

C

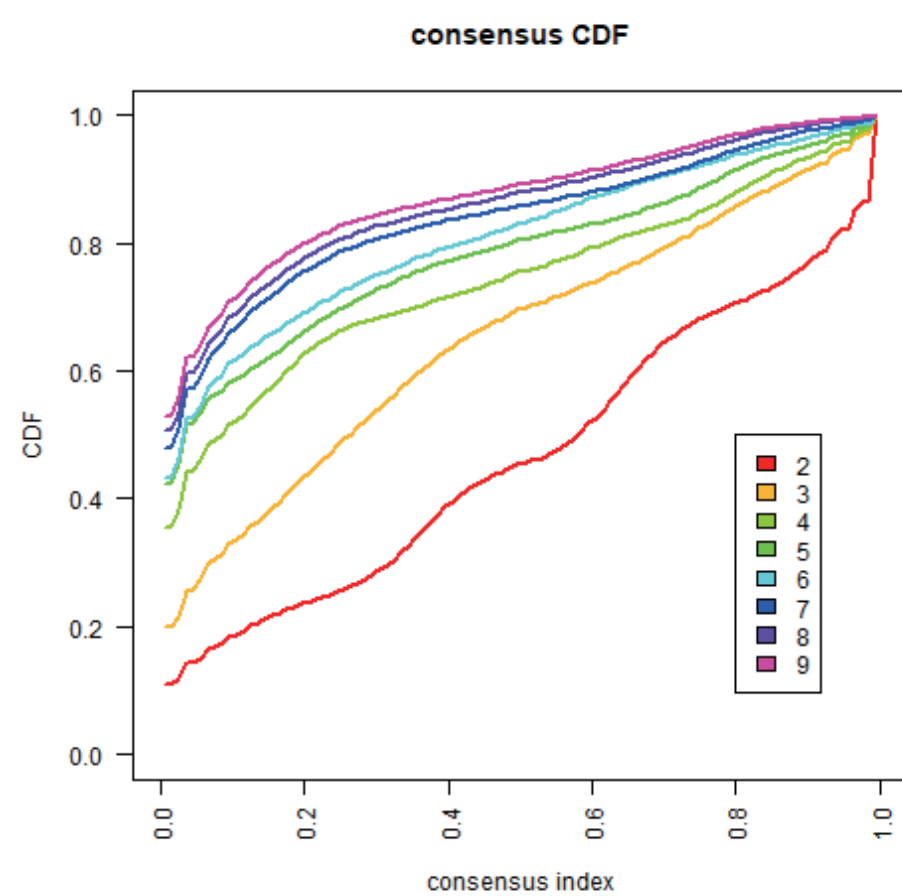

D

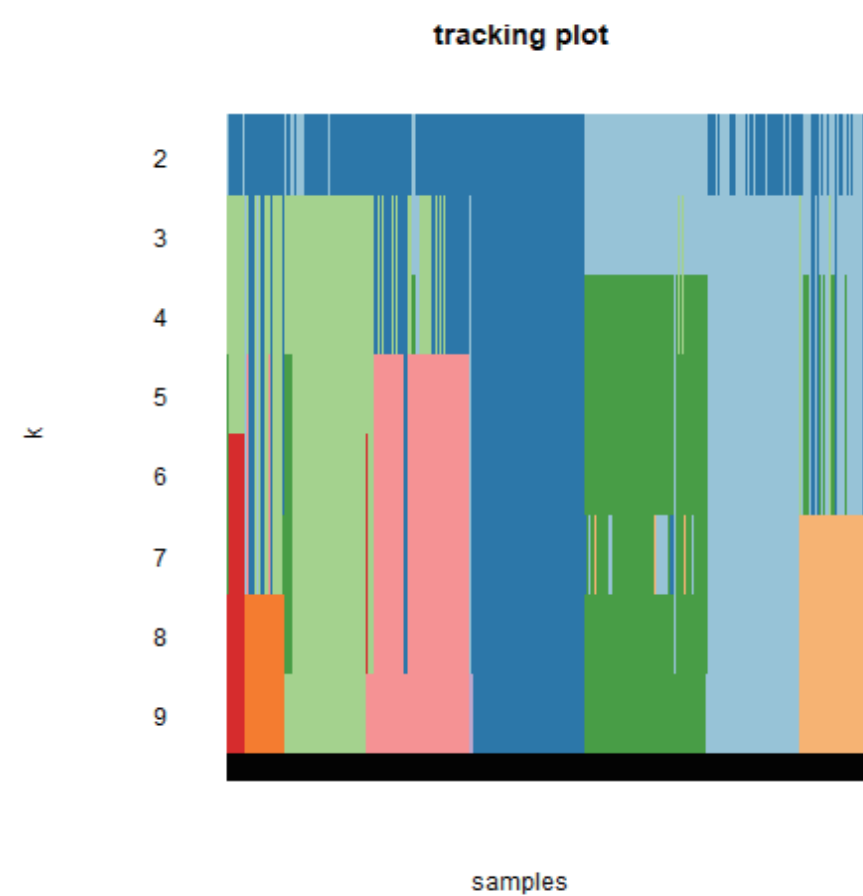

Supplement: Supplementary file 2 — Additional file 2: Figure S1.Boxplot of the expression of PAM genes in tumor and normal sample on the TCGA cohort.Heatmap of significant different PAM genes in tumor and normal sample..GO enrichment analysis for PAM genes.KEGG enrichment analysis for PAM genes.Forest plot of prognostic gene with Univariate cox regression analysis.Protein–protein interactionfor PAM-related genes.Venn diagram showing PAM genes after intersection of datasets. Figure S2. Survival prognostic analysis of each PAM gene with high and low expression using Kaplan–Meier analysis. Figure S3.Consensus clustering of 55 PAM genes matrix for k = 3 of 1224 patients in the TCGA cohort combine the GEO cohort.Determine the relevant CDF curve and Tracking plot of Consensus clustering. Figure S4.Consensus clustering of 328 PAM prognostic genes in the meta dataset.GO enrichment analysis and KEGG enrichment analysis of 328 PAM prognostic genes.Expression heatmap of 328 PAM prognostic genes in geneCluster A and B subgroups.Forest plots for univariate and multivariate cox analysis of PAMscore. Figure S5. Survival analysis of high and low score subgroups of PAMscore for different genders, different T, N, M stages and AJCC stages in CRC patients. Figure S6.RNA expression of ACAT2, SPHK1, SNED1, KPNA2, BZW2 and KIF15 in tumor and normal tissues in TCGA dataset.IHC cell staining intensity in normal and tumor tissues of the CRC cohort. Figure S7.Expression levels of marker genes in the 6 cell types.Expression levels of marker genes in high and low cell groups. [file 12935_2023_2892_MOESM2_ESM.zip › Supplementary Material 2/S3.pdf]

A

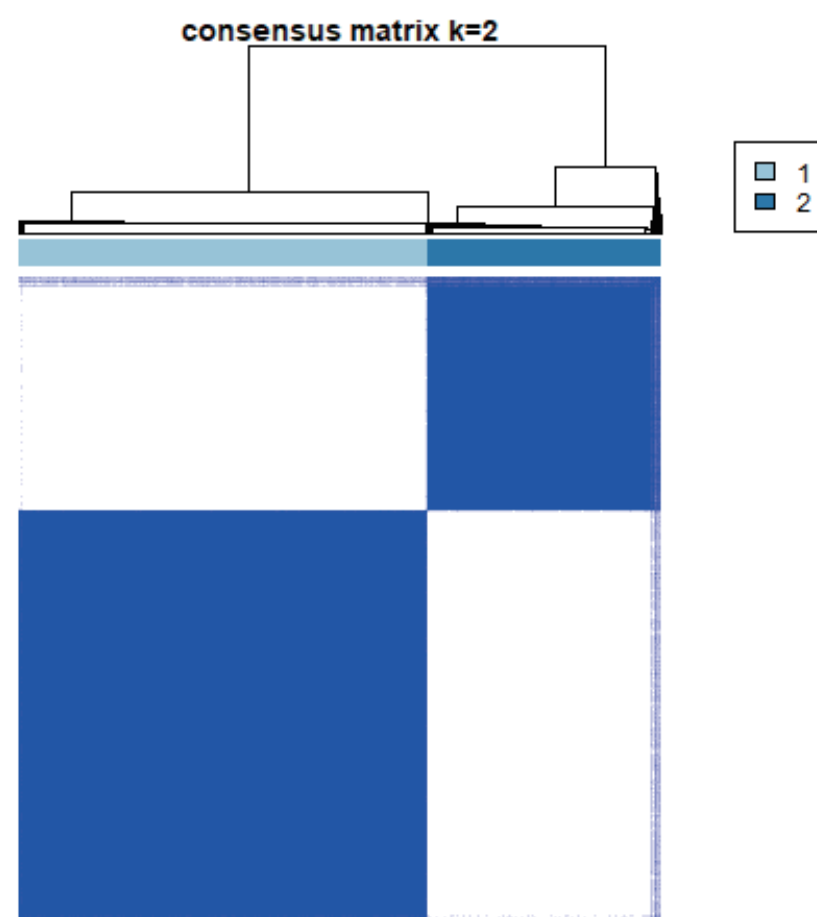

B

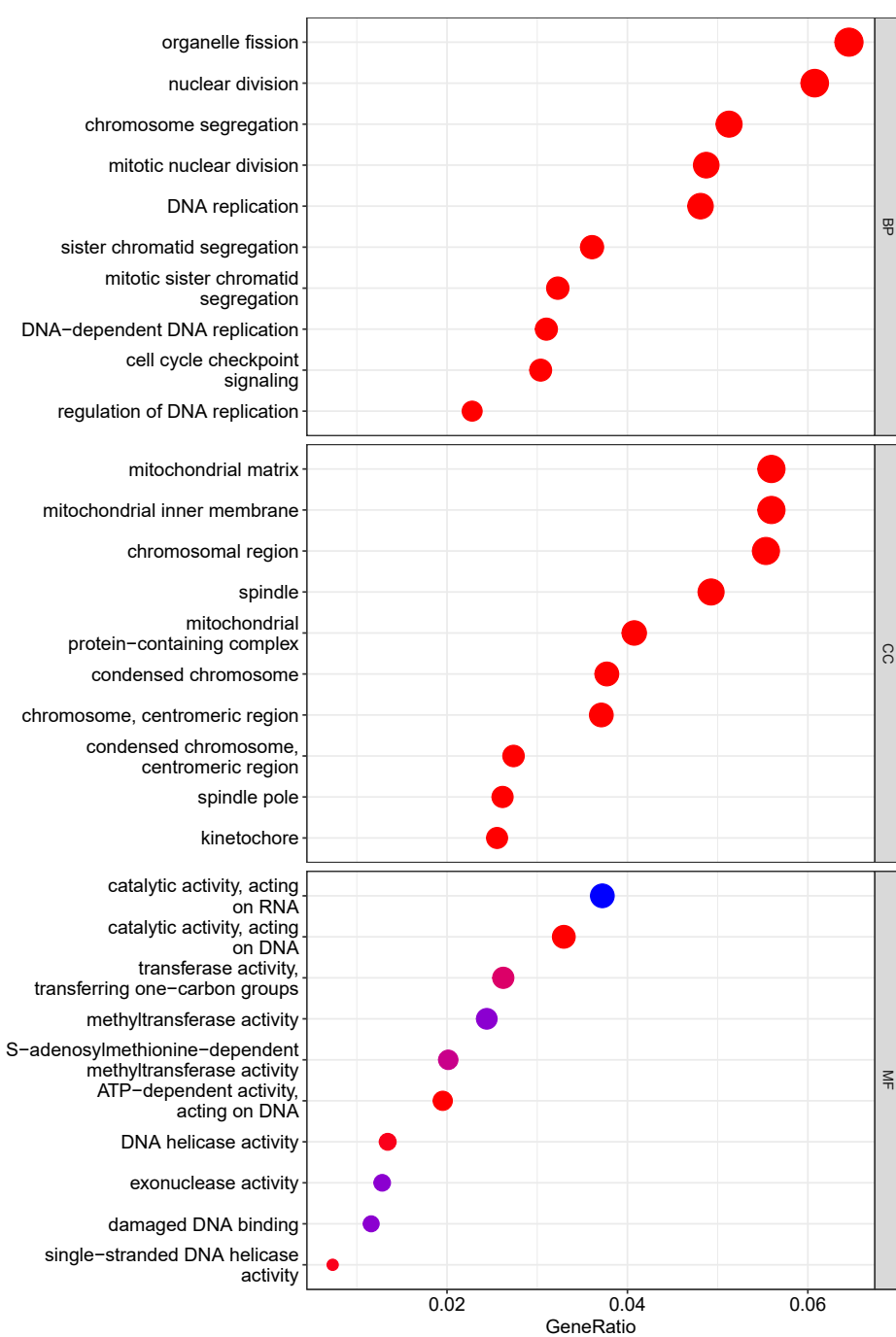

C

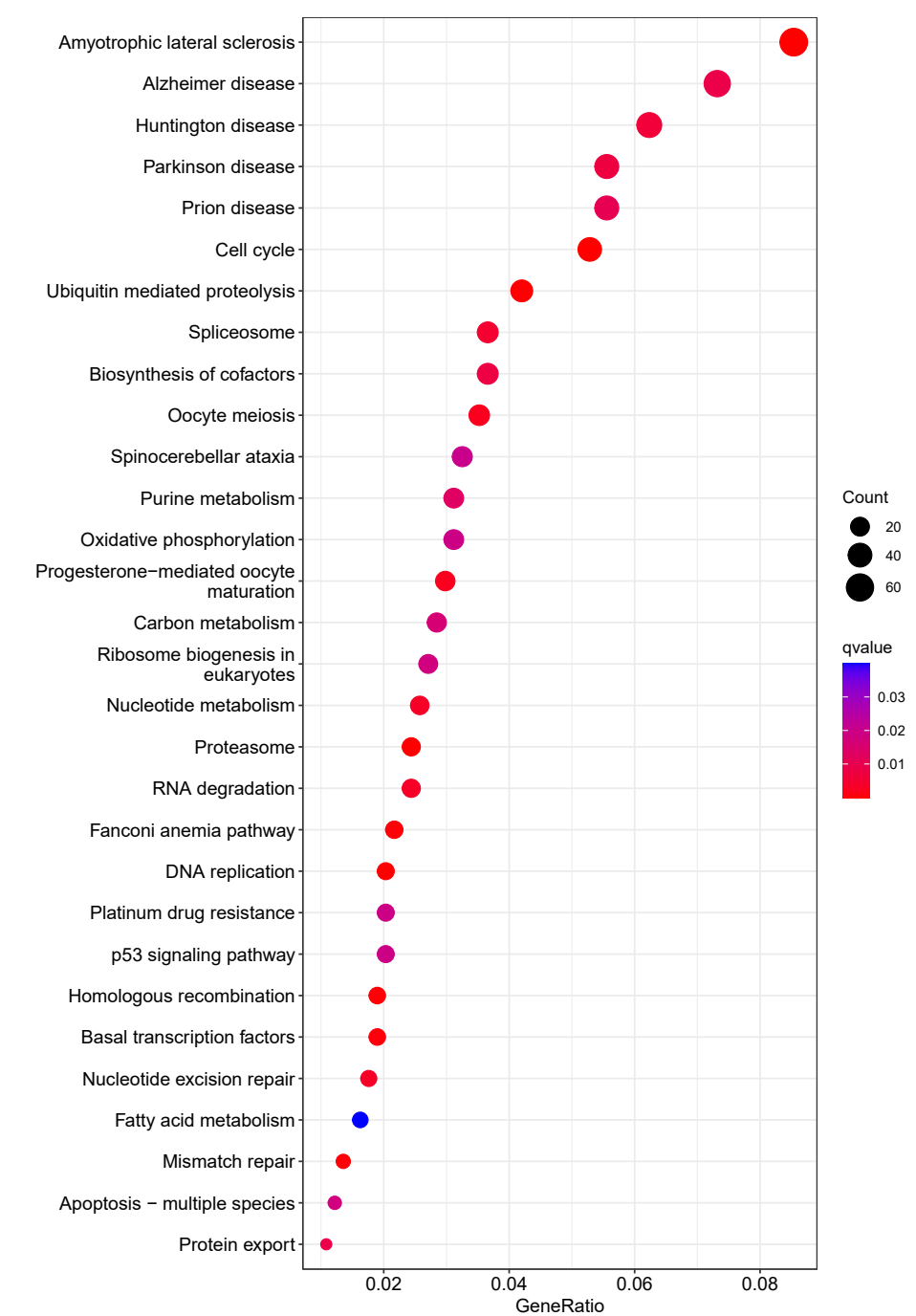

D

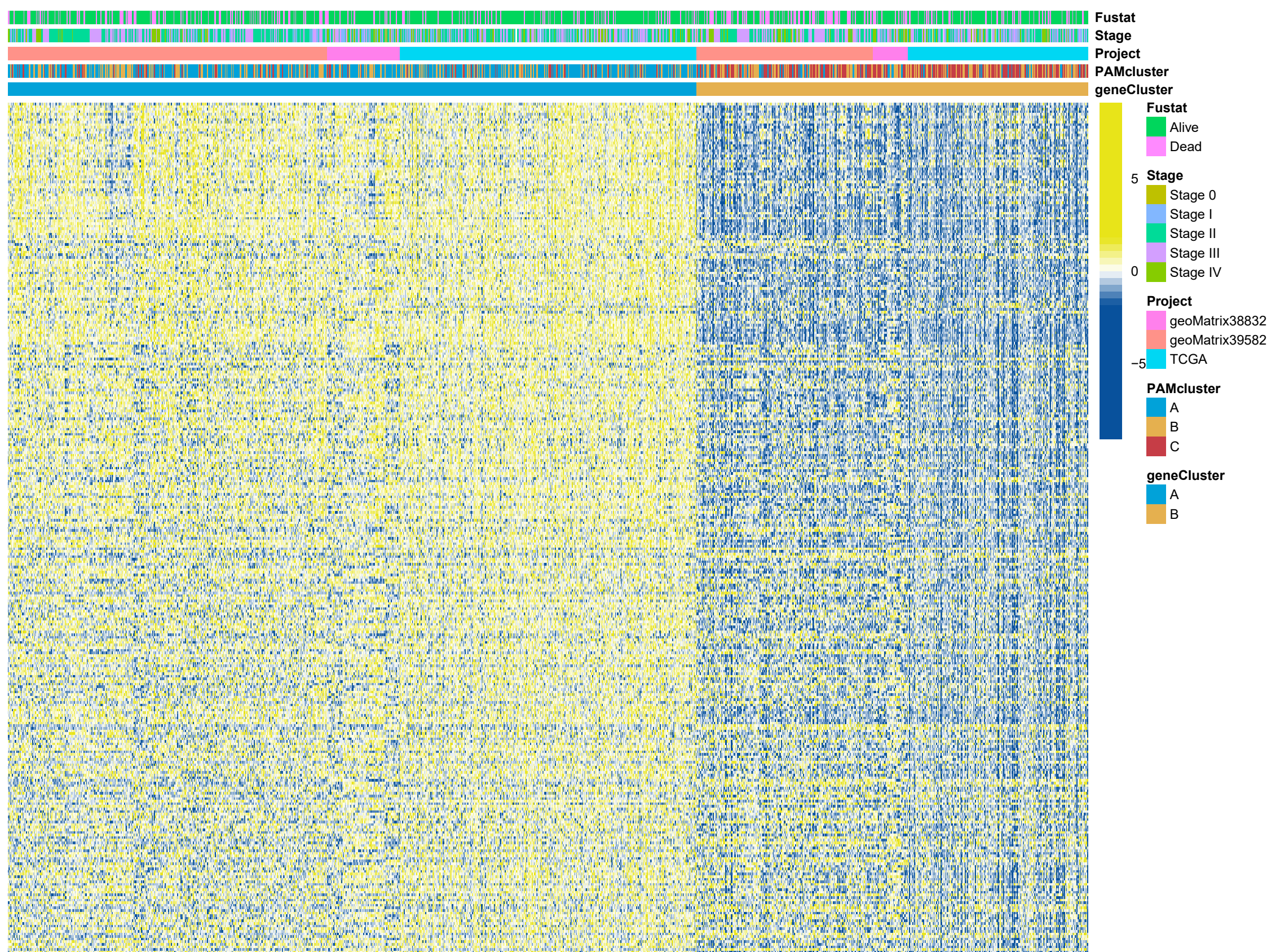

E

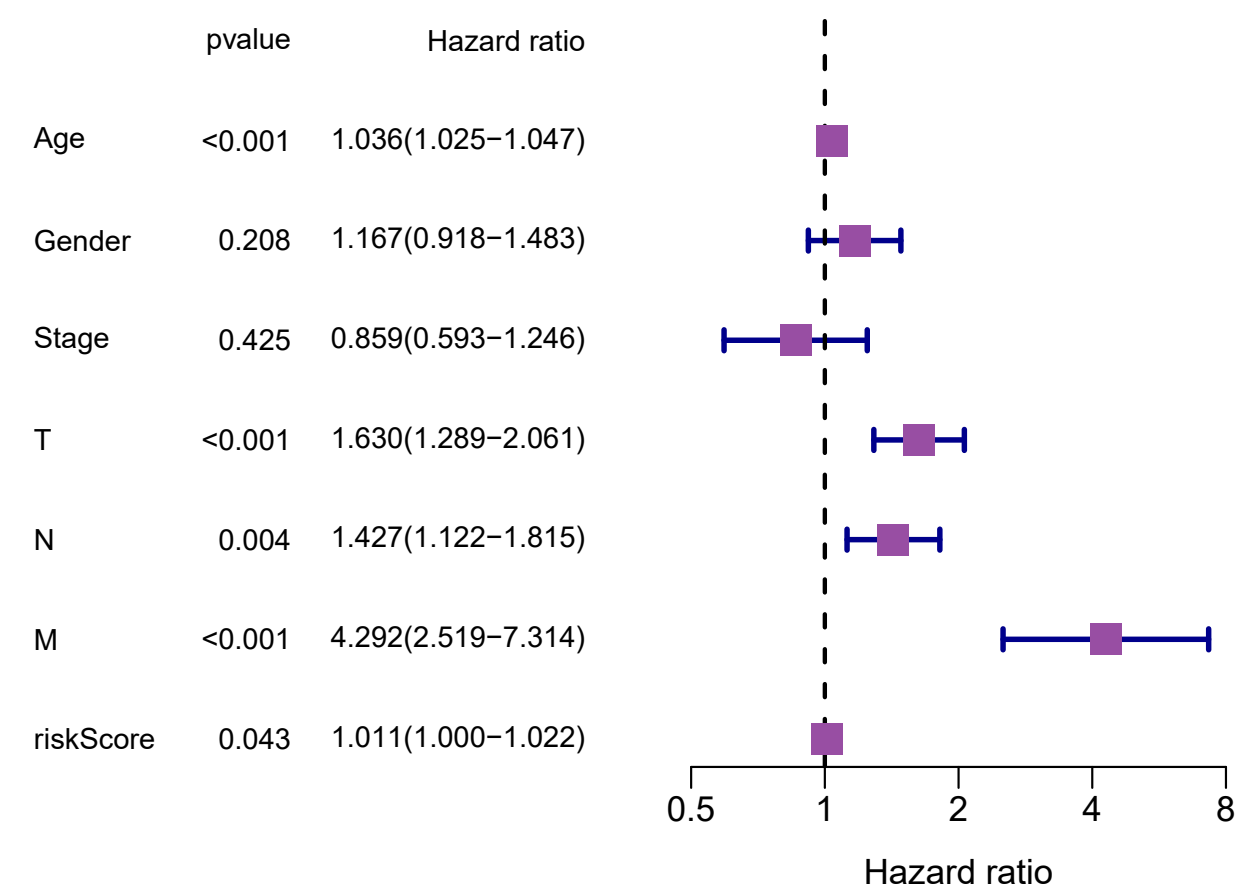

F

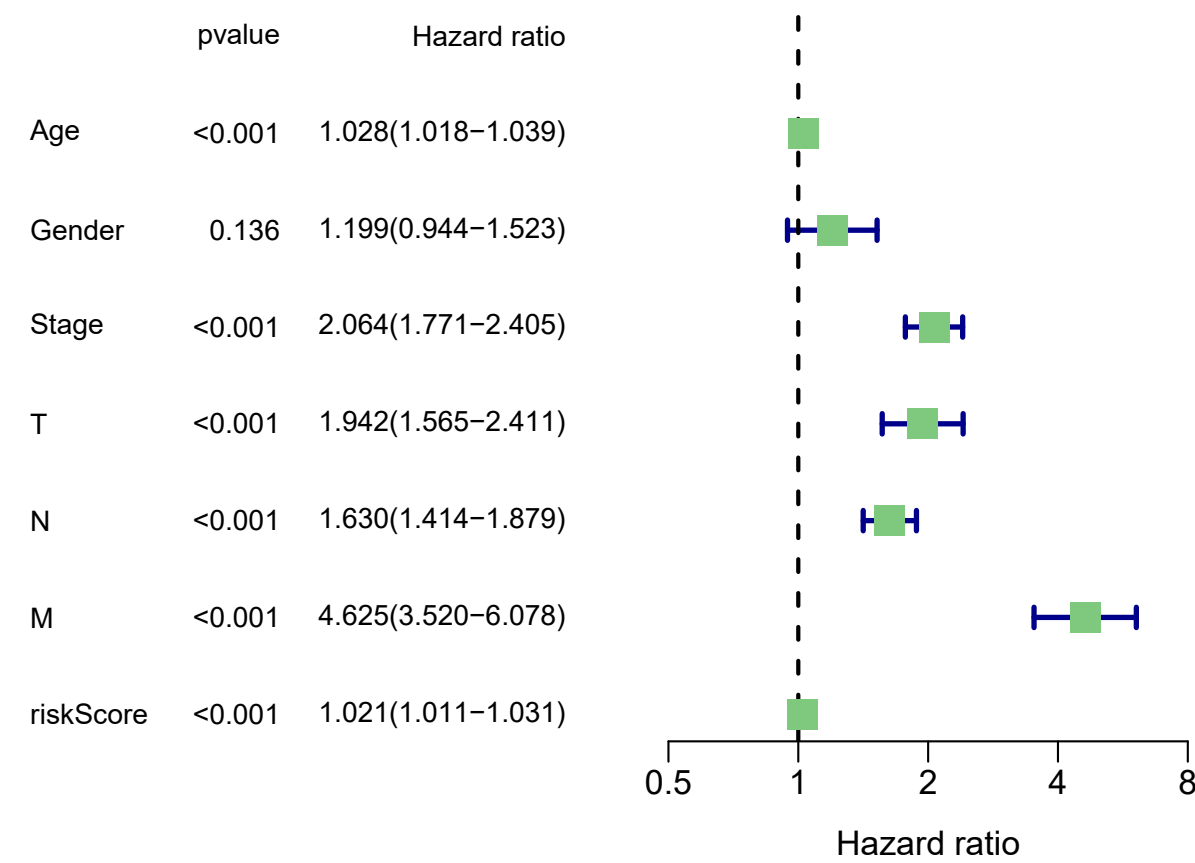

Supplement: Supplementary file 2 — Additional file 2: Figure S1.Boxplot of the expression of PAM genes in tumor and normal sample on the TCGA cohort.Heatmap of significant different PAM genes in tumor and normal sample..GO enrichment analysis for PAM genes.KEGG enrichment analysis for PAM genes.Forest plot of prognostic gene with Univariate cox regression analysis.Protein–protein interactionfor PAM-related genes.Venn diagram showing PAM genes after intersection of datasets. Figure S2. Survival prognostic analysis of each PAM gene with high and low expression using Kaplan–Meier analysis. Figure S3.Consensus clustering of 55 PAM genes matrix for k = 3 of 1224 patients in the TCGA cohort combine the GEO cohort.Determine the relevant CDF curve and Tracking plot of Consensus clustering. Figure S4.Consensus clustering of 328 PAM prognostic genes in the meta dataset.GO enrichment analysis and KEGG enrichment analysis of 328 PAM prognostic genes.Expression heatmap of 328 PAM prognostic genes in geneCluster A and B subgroups.Forest plots for univariate and multivariate cox analysis of PAMscore. Figure S5. Survival analysis of high and low score subgroups of PAMscore for different genders, different T, N, M stages and AJCC stages in CRC patients. Figure S6.RNA expression of ACAT2, SPHK1, SNED1, KPNA2, BZW2 and KIF15 in tumor and normal tissues in TCGA dataset.IHC cell staining intensity in normal and tumor tissues of the CRC cohort. Figure S7.Expression levels of marker genes in the 6 cell types.Expression levels of marker genes in high and low cell groups. [file 12935_2023_2892_MOESM2_ESM.zip › Supplementary Material 2/S4.pdf]

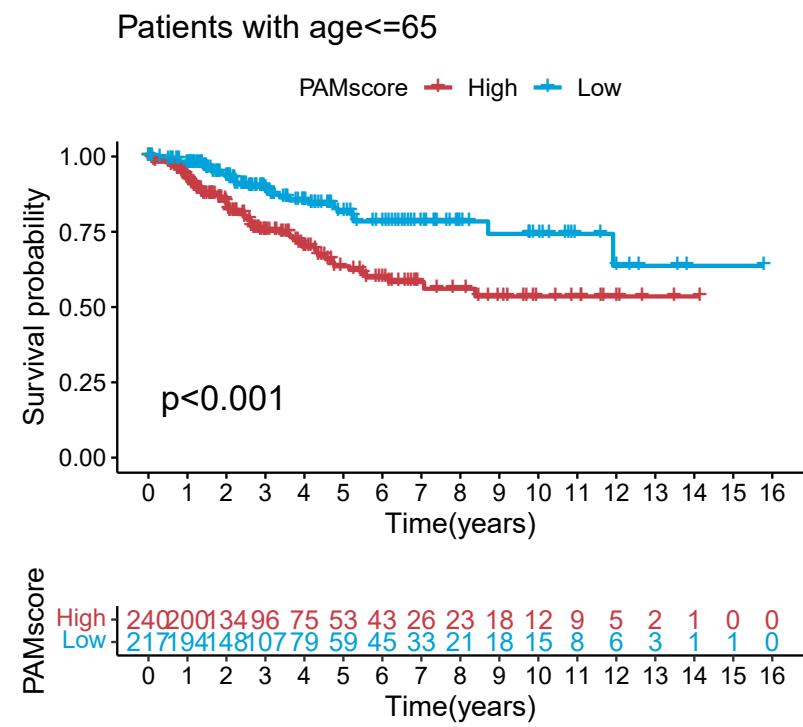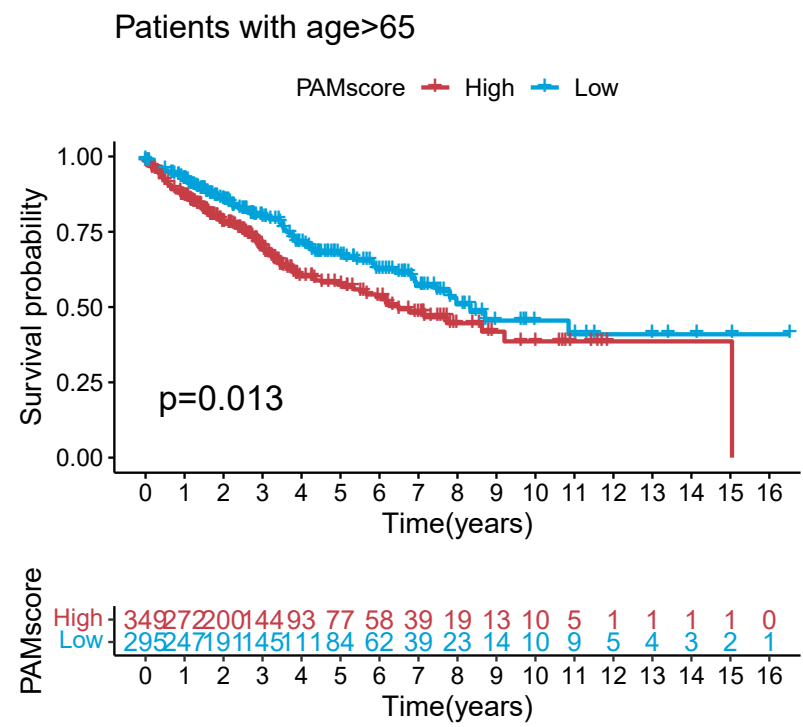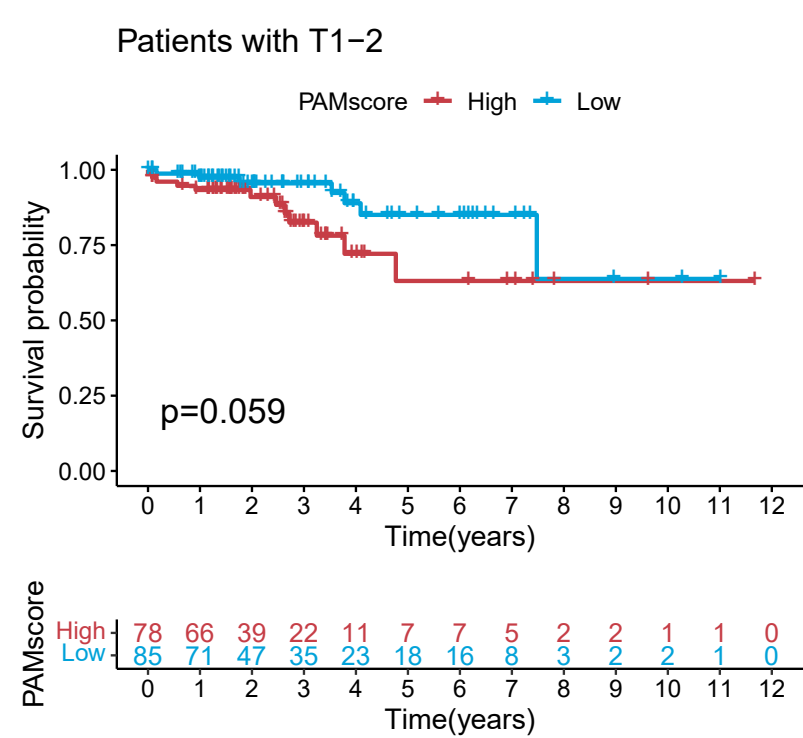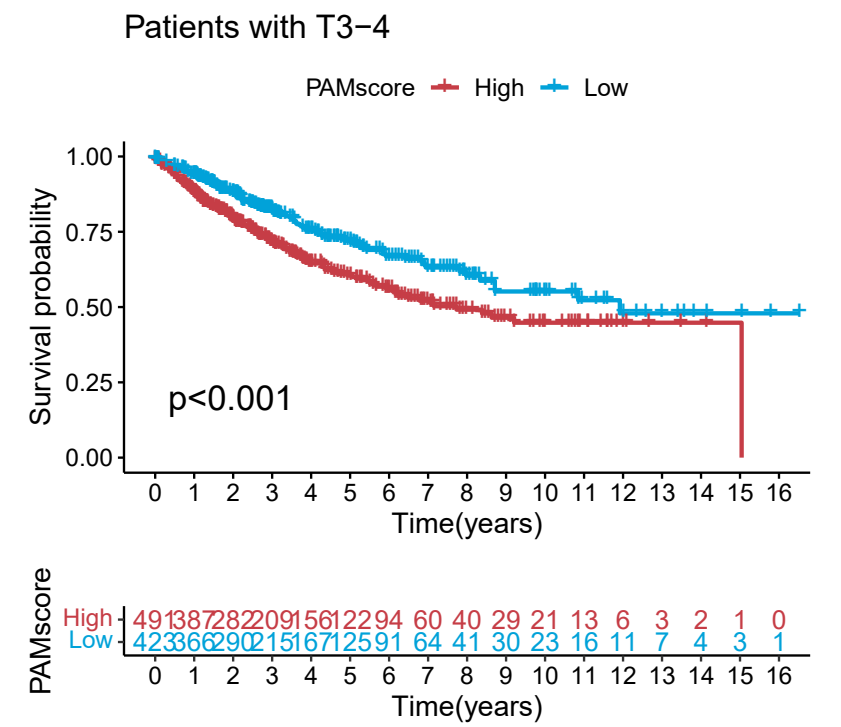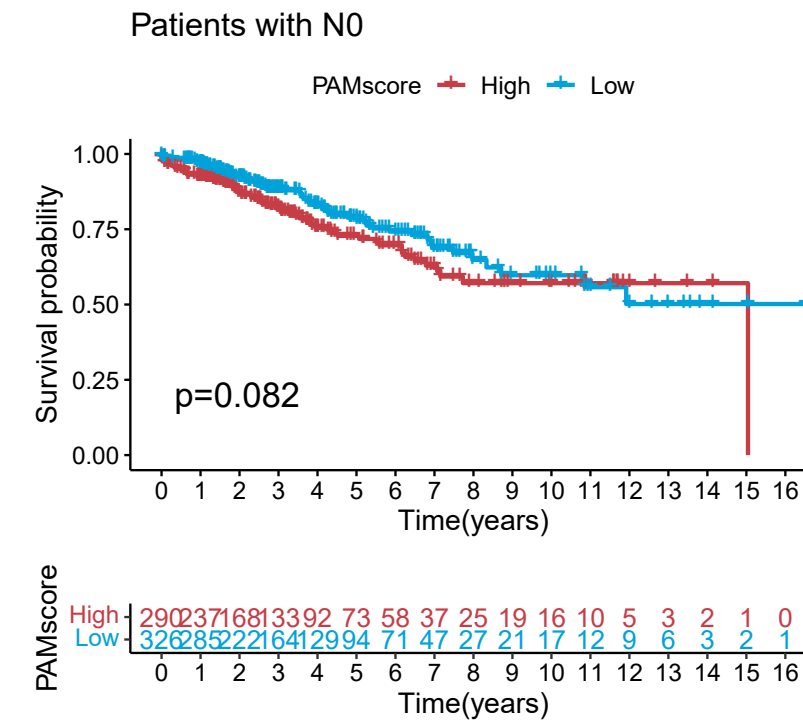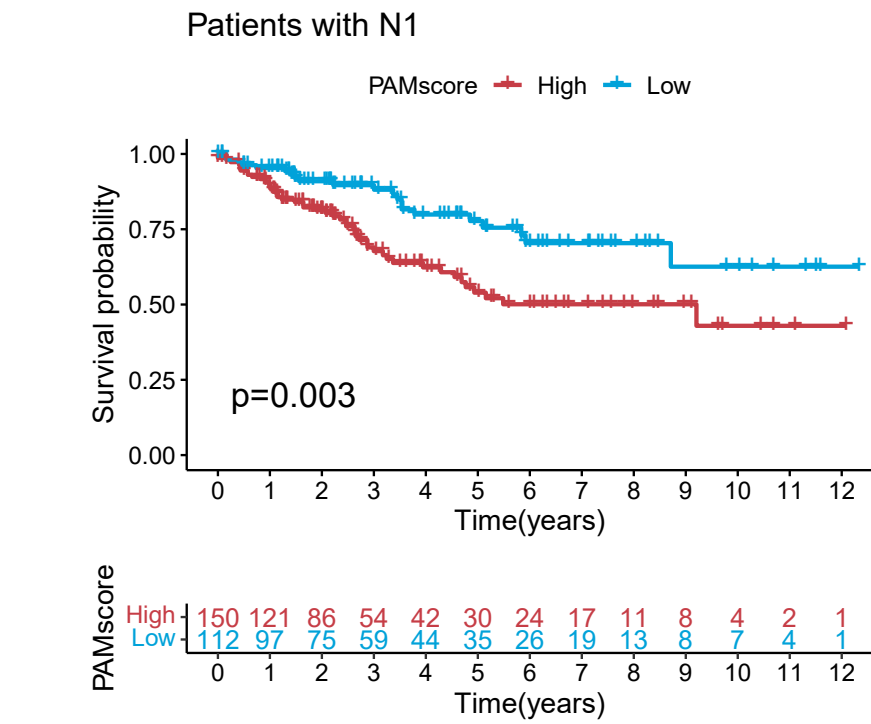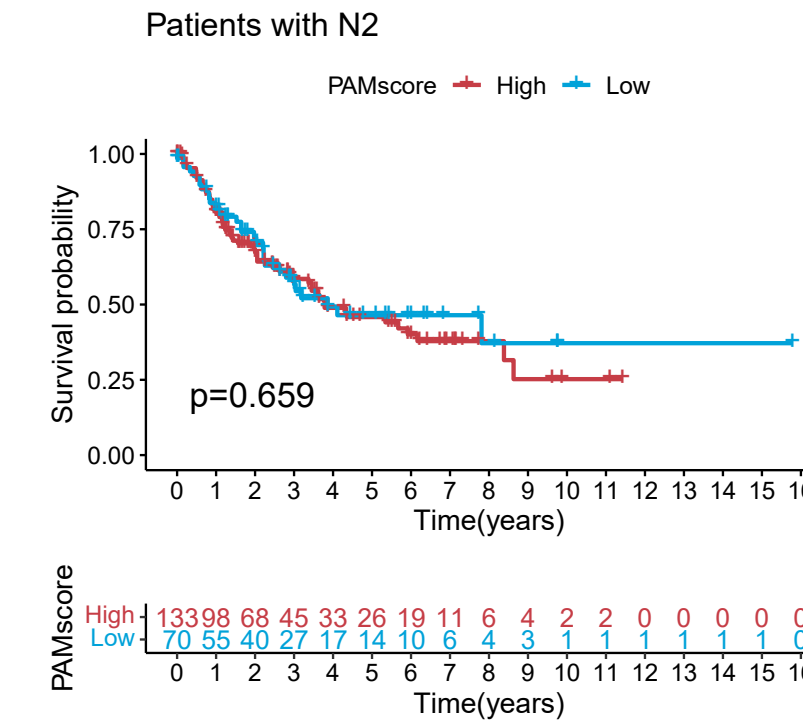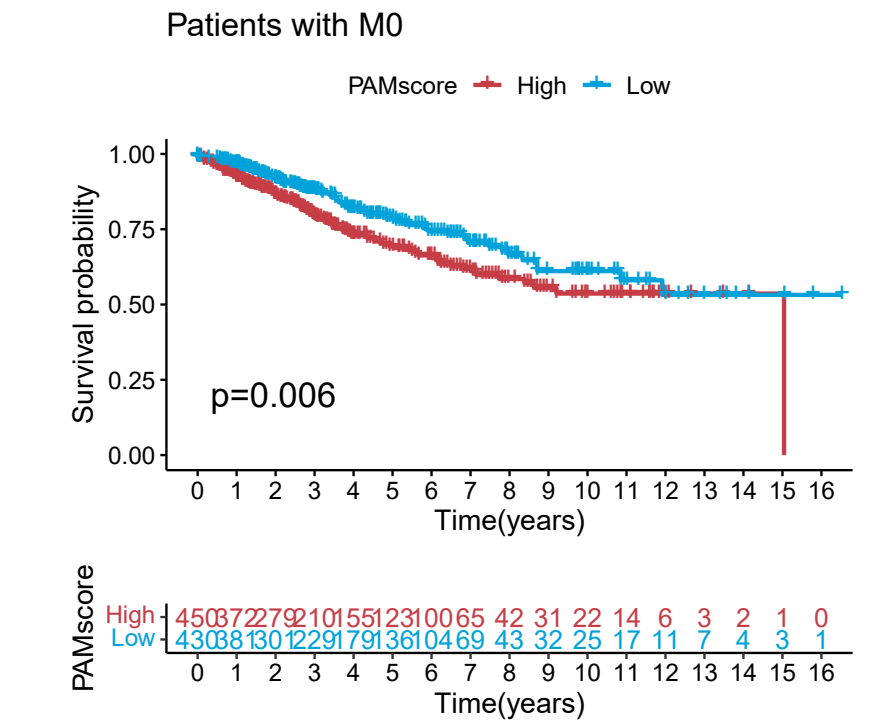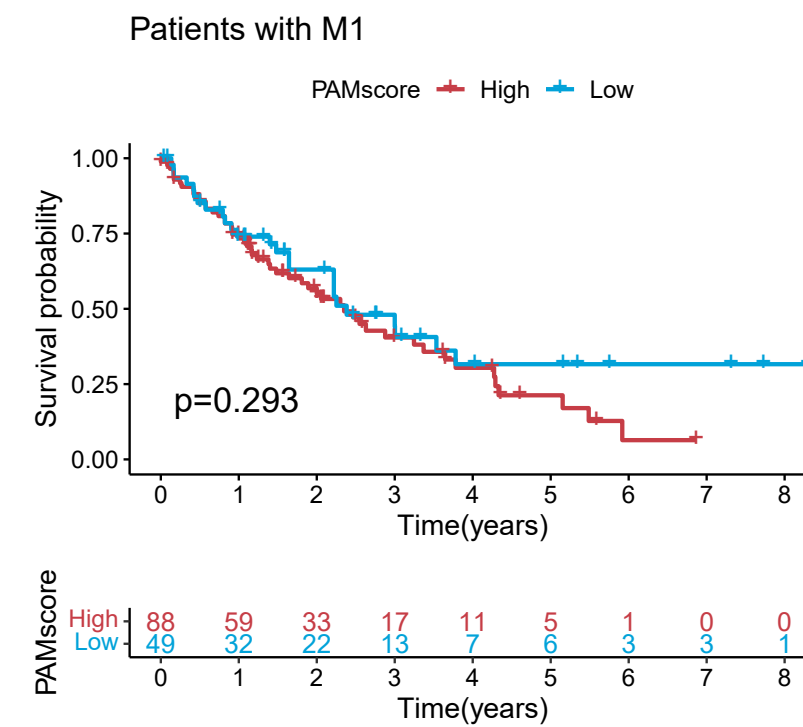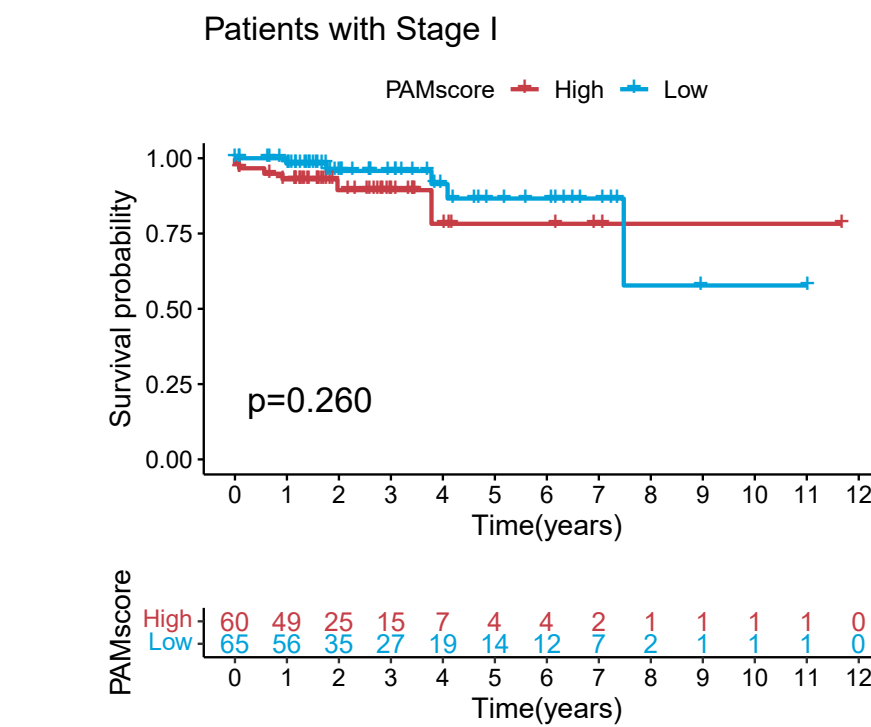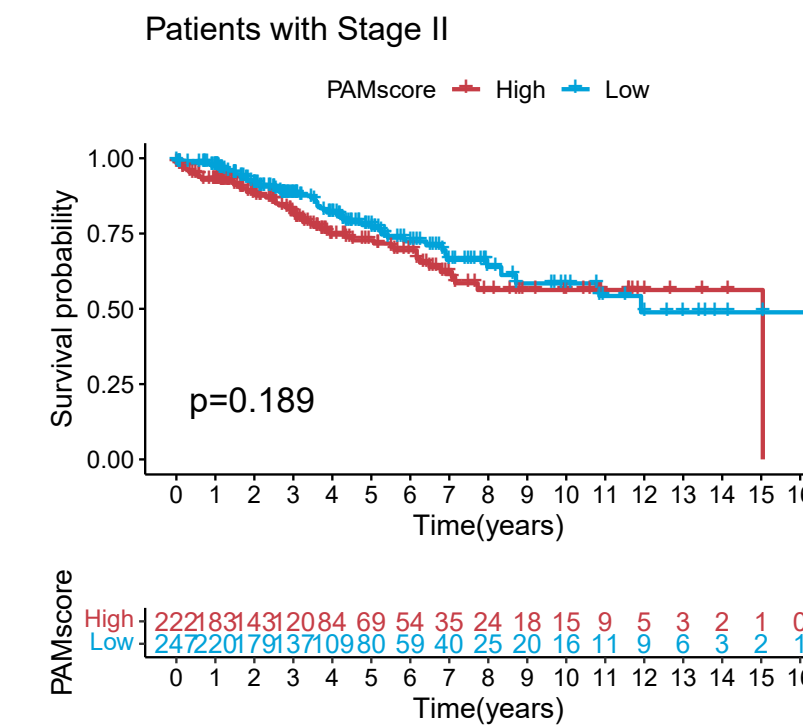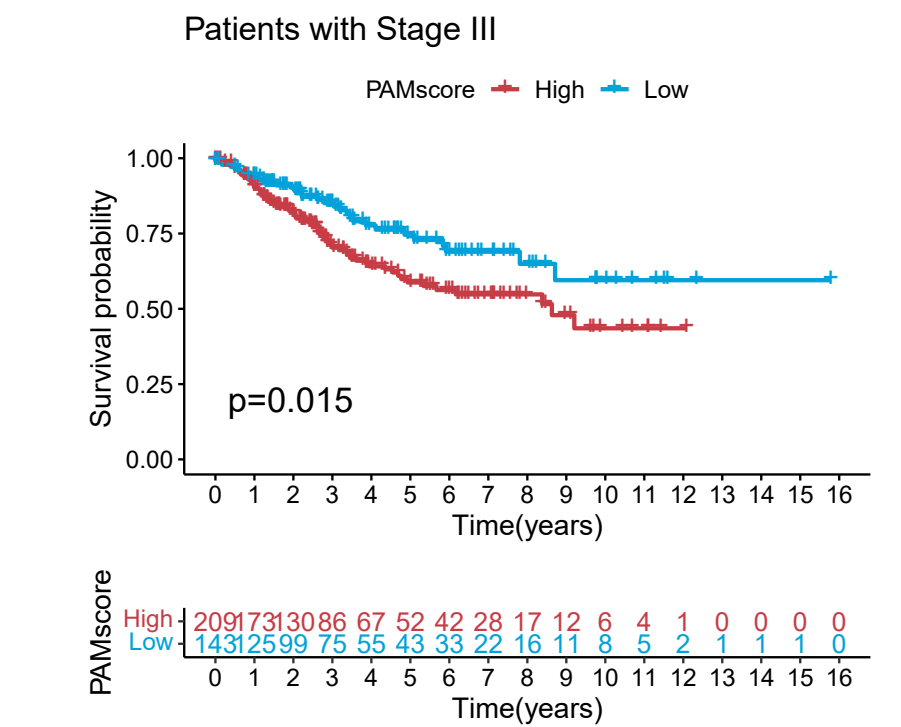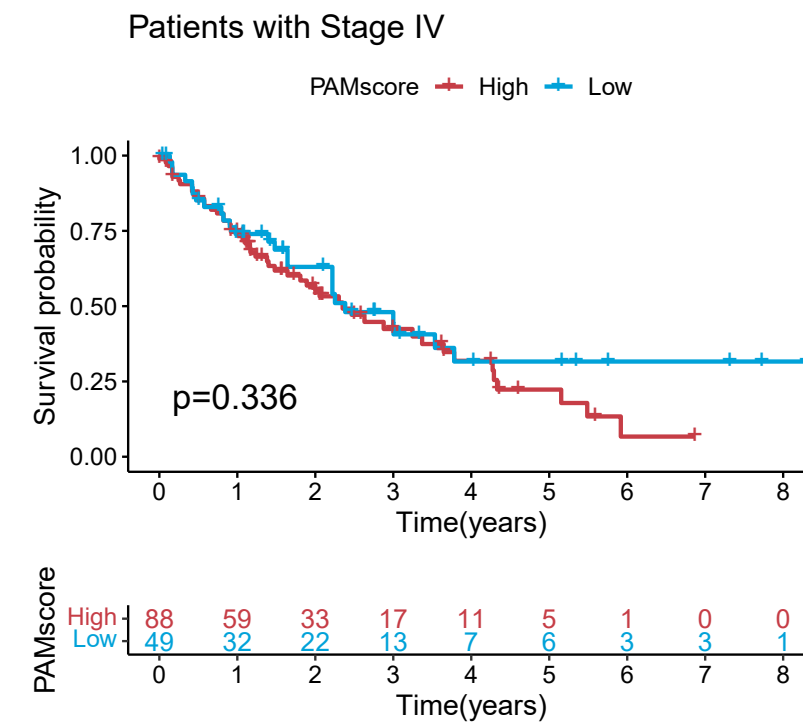

Supplement: Supplementary file 2 — Additional file 2: Figure S1.Boxplot of the expression of PAM genes in tumor and normal sample on the TCGA cohort.Heatmap of significant different PAM genes in tumor and normal sample..GO enrichment analysis for PAM genes.KEGG enrichment analysis for PAM genes.Forest plot of prognostic gene with Univariate cox regression analysis.Protein–protein interactionfor PAM-related genes.Venn diagram showing PAM genes after intersection of datasets. Figure S2. Survival prognostic analysis of each PAM gene with high and low expression using Kaplan–Meier analysis. Figure S3.Consensus clustering of 55 PAM genes matrix for k = 3 of 1224 patients in the TCGA cohort combine the GEO cohort.Determine the relevant CDF curve and Tracking plot of Consensus clustering. Figure S4.Consensus clustering of 328 PAM prognostic genes in the meta dataset.GO enrichment analysis and KEGG enrichment analysis of 328 PAM prognostic genes.Expression heatmap of 328 PAM prognostic genes in geneCluster A and B subgroups.Forest plots for univariate and multivariate cox analysis of PAMscore. Figure S5. Survival analysis of high and low score subgroups of PAMscore for different genders, different T, N, M stages and AJCC stages in CRC patients. Figure S6.RNA expression of ACAT2, SPHK1, SNED1, KPNA2, BZW2 and KIF15 in tumor and normal tissues in TCGA dataset.IHC cell staining intensity in normal and tumor tissues of the CRC cohort. Figure S7.Expression levels of marker genes in the 6 cell types.Expression levels of marker genes in high and low cell groups. [file 12935_2023_2892_MOESM2_ESM.zip › Supplementary Material 2/S5.pdf]

A

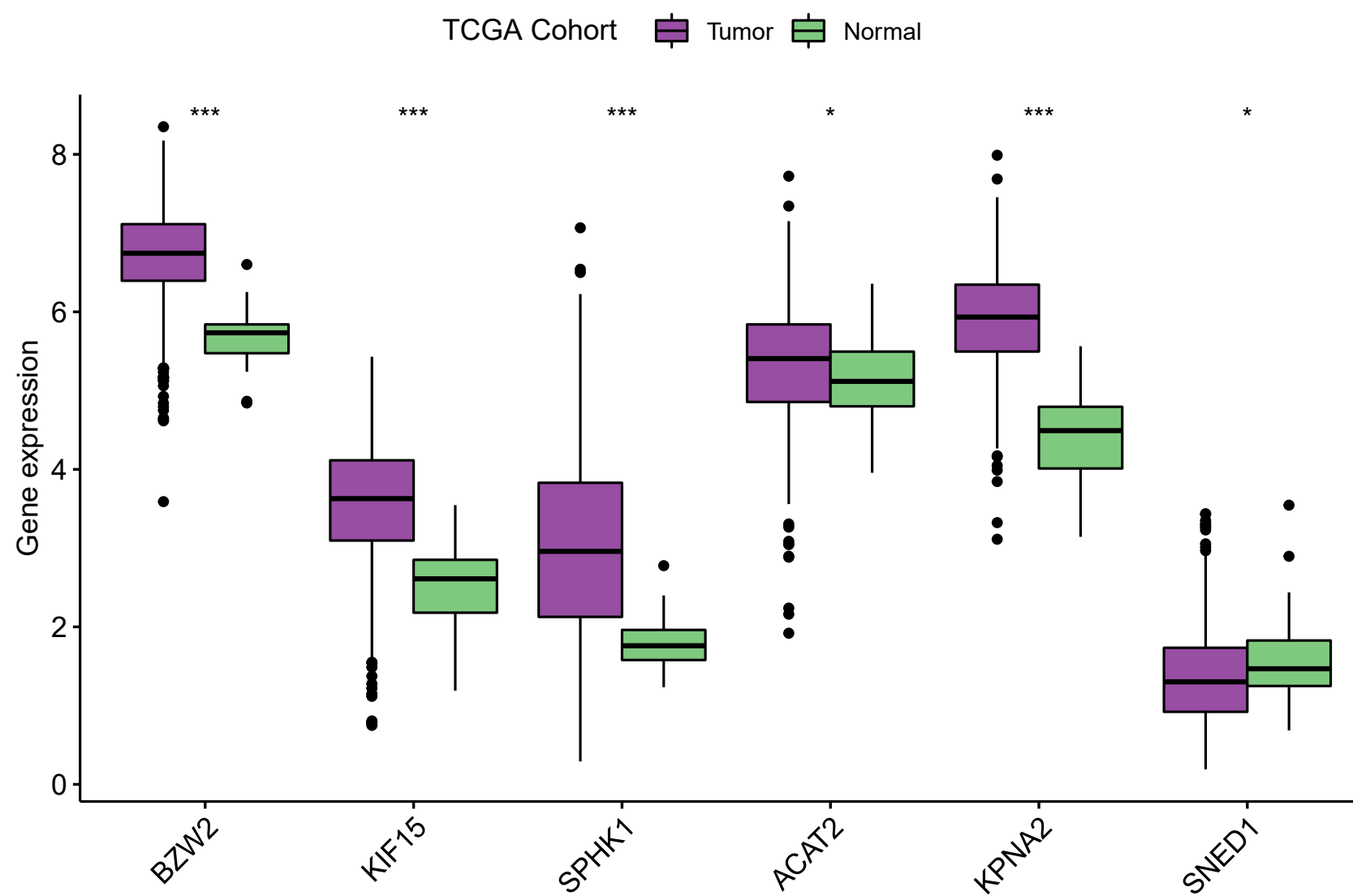

B

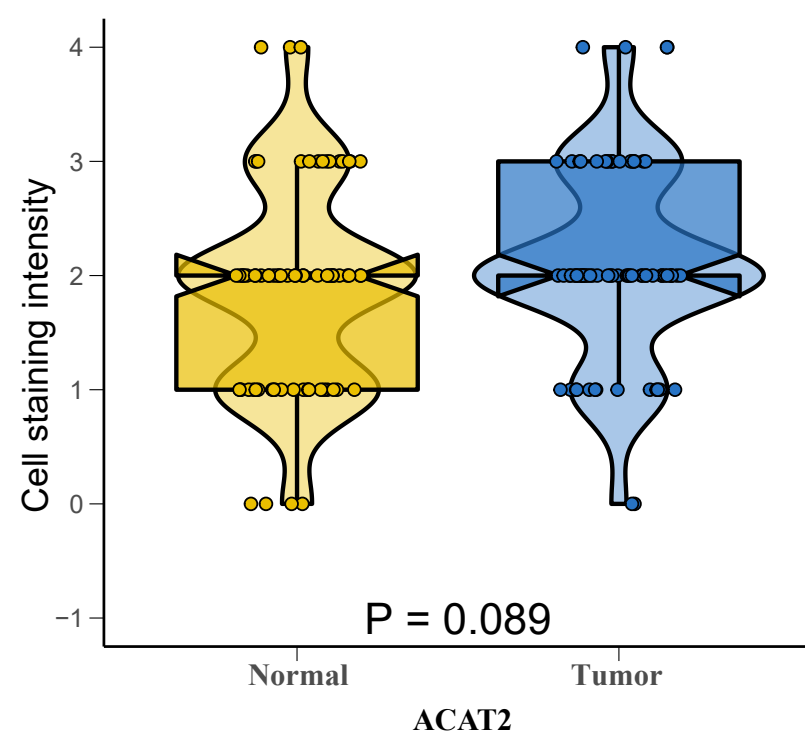

C

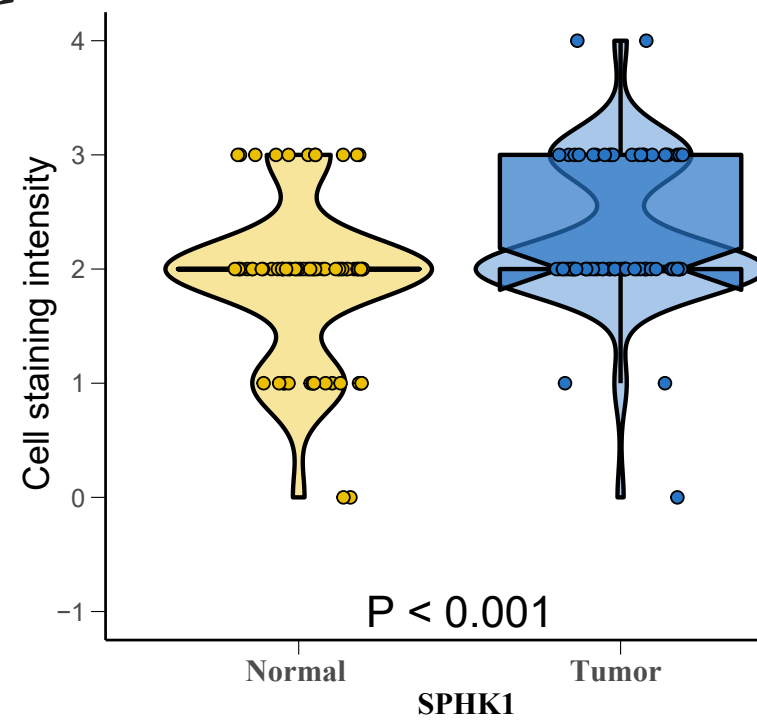

D

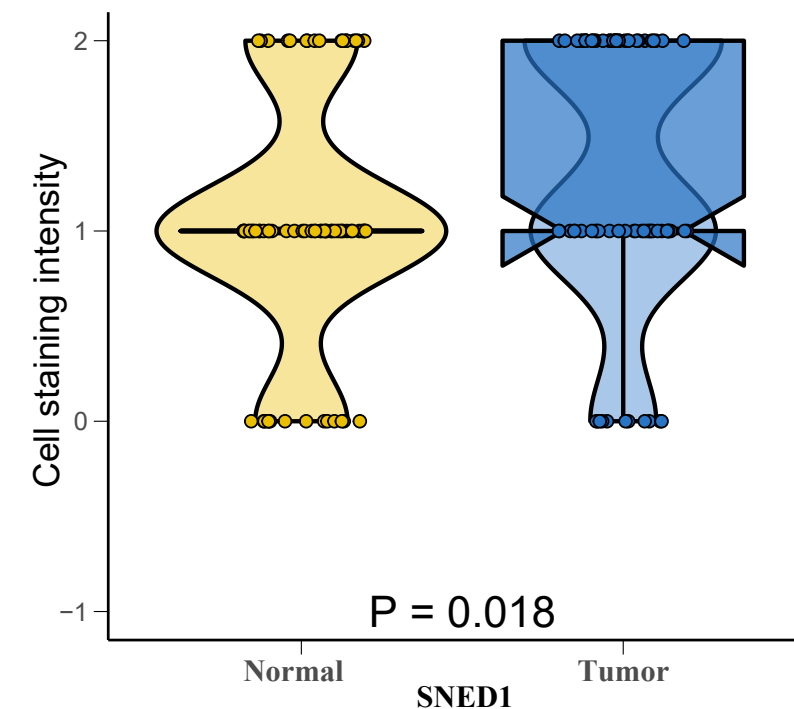

E

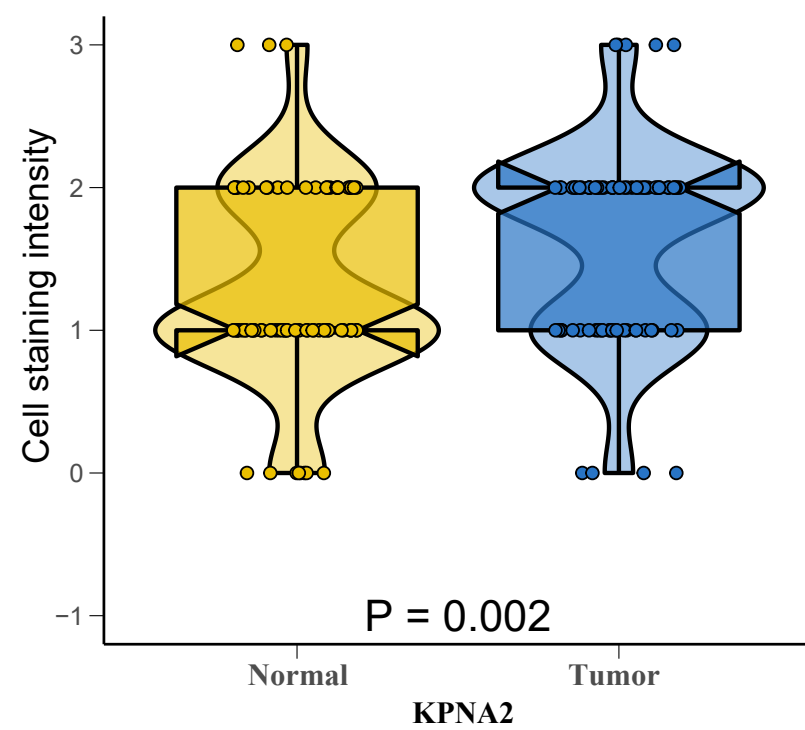

F

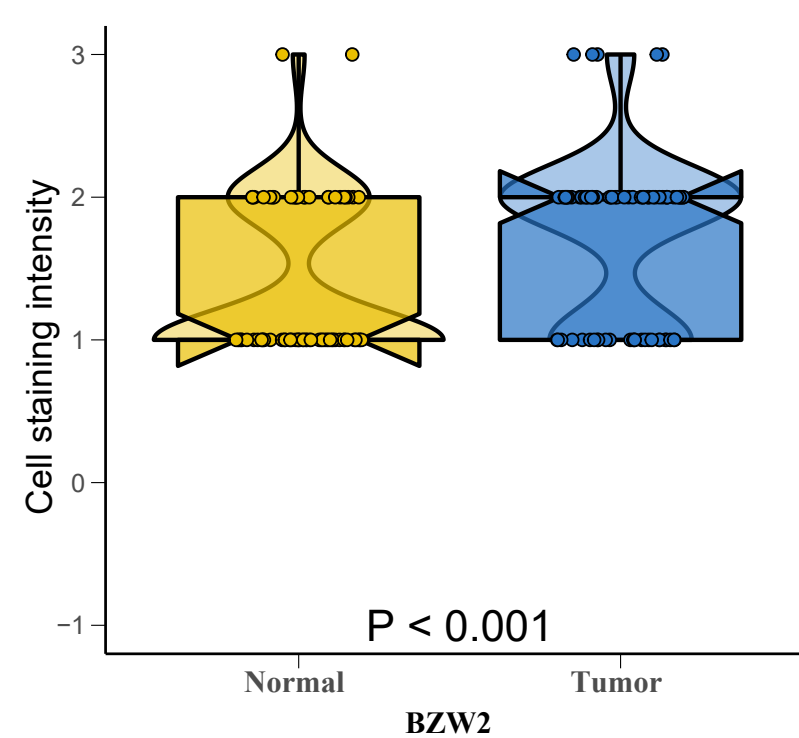

G

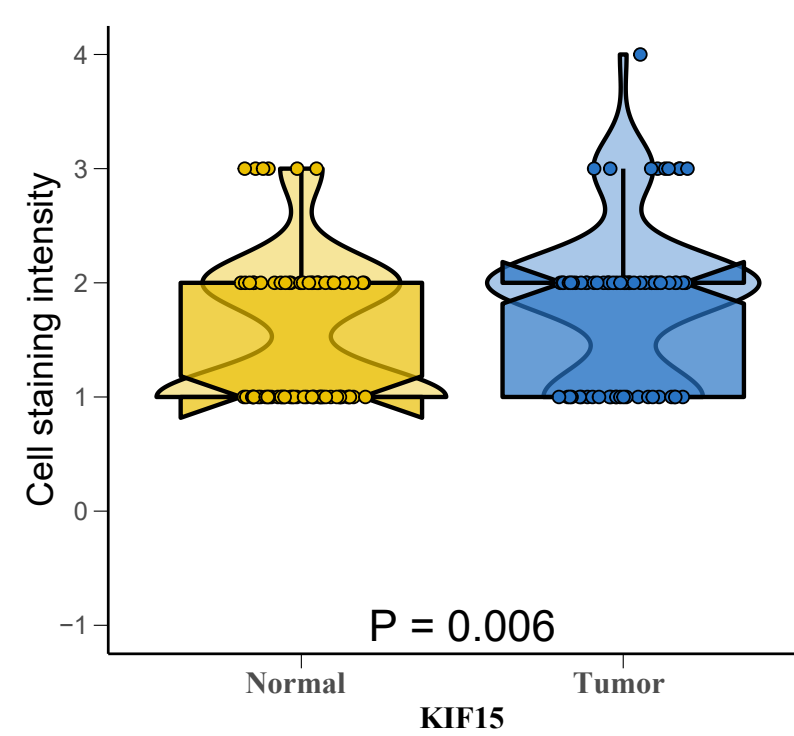

Supplement: Supplementary file 2 — Additional file 2: Figure S1.Boxplot of the expression of PAM genes in tumor and normal sample on the TCGA cohort.Heatmap of significant different PAM genes in tumor and normal sample..GO enrichment analysis for PAM genes.KEGG enrichment analysis for PAM genes.Forest plot of prognostic gene with Univariate cox regression analysis.Protein–protein interactionfor PAM-related genes.Venn diagram showing PAM genes after intersection of datasets. Figure S2. Survival prognostic analysis of each PAM gene with high and low expression using Kaplan–Meier analysis. Figure S3.Consensus clustering of 55 PAM genes matrix for k = 3 of 1224 patients in the TCGA cohort combine the GEO cohort.Determine the relevant CDF curve and Tracking plot of Consensus clustering. Figure S4.Consensus clustering of 328 PAM prognostic genes in the meta dataset.GO enrichment analysis and KEGG enrichment analysis of 328 PAM prognostic genes.Expression heatmap of 328 PAM prognostic genes in geneCluster A and B subgroups.Forest plots for univariate and multivariate cox analysis of PAMscore. Figure S5. Survival analysis of high and low score subgroups of PAMscore for different genders, different T, N, M stages and AJCC stages in CRC patients. Figure S6.RNA expression of ACAT2, SPHK1, SNED1, KPNA2, BZW2 and KIF15 in tumor and normal tissues in TCGA dataset.IHC cell staining intensity in normal and tumor tissues of the CRC cohort. Figure S7.Expression levels of marker genes in the 6 cell types.Expression levels of marker genes in high and low cell groups. [file 12935_2023_2892_MOESM2_ESM.zip › Supplementary Material 2/S6.pdf]

A

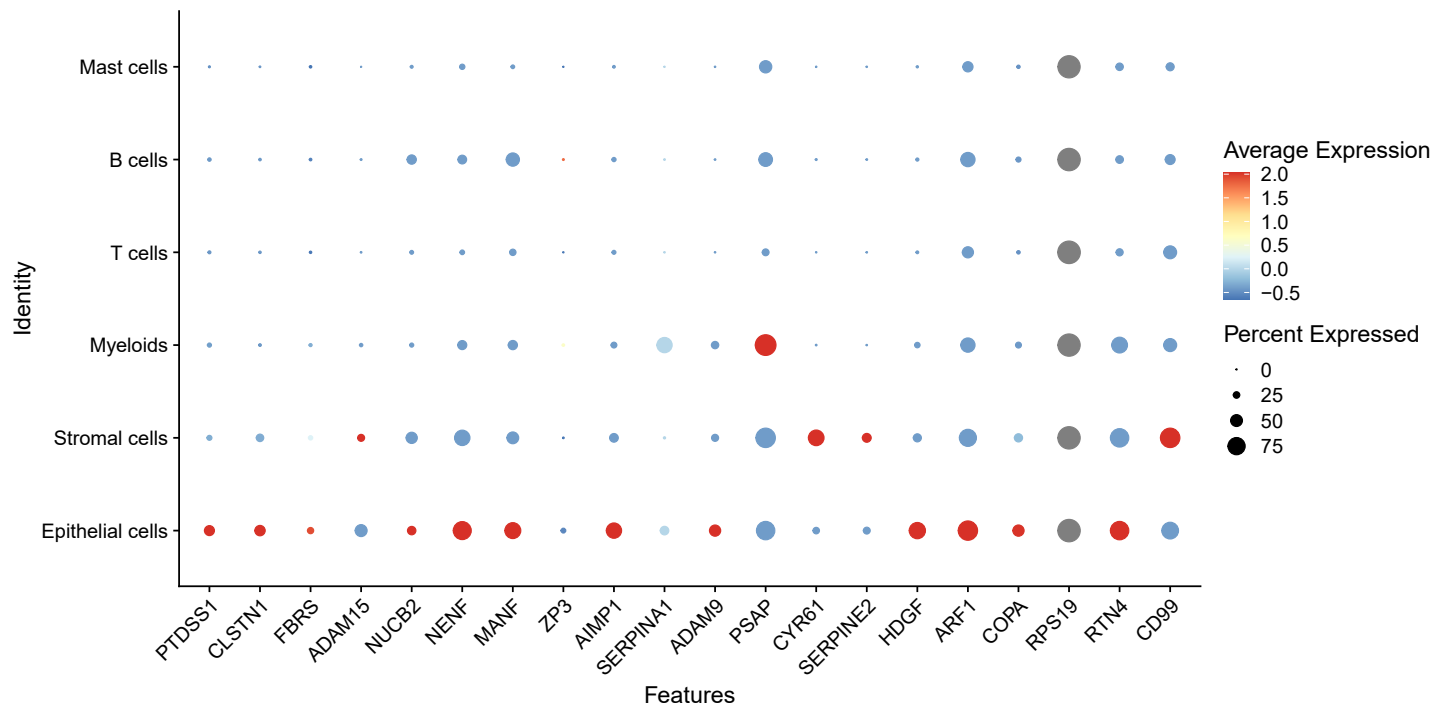

B

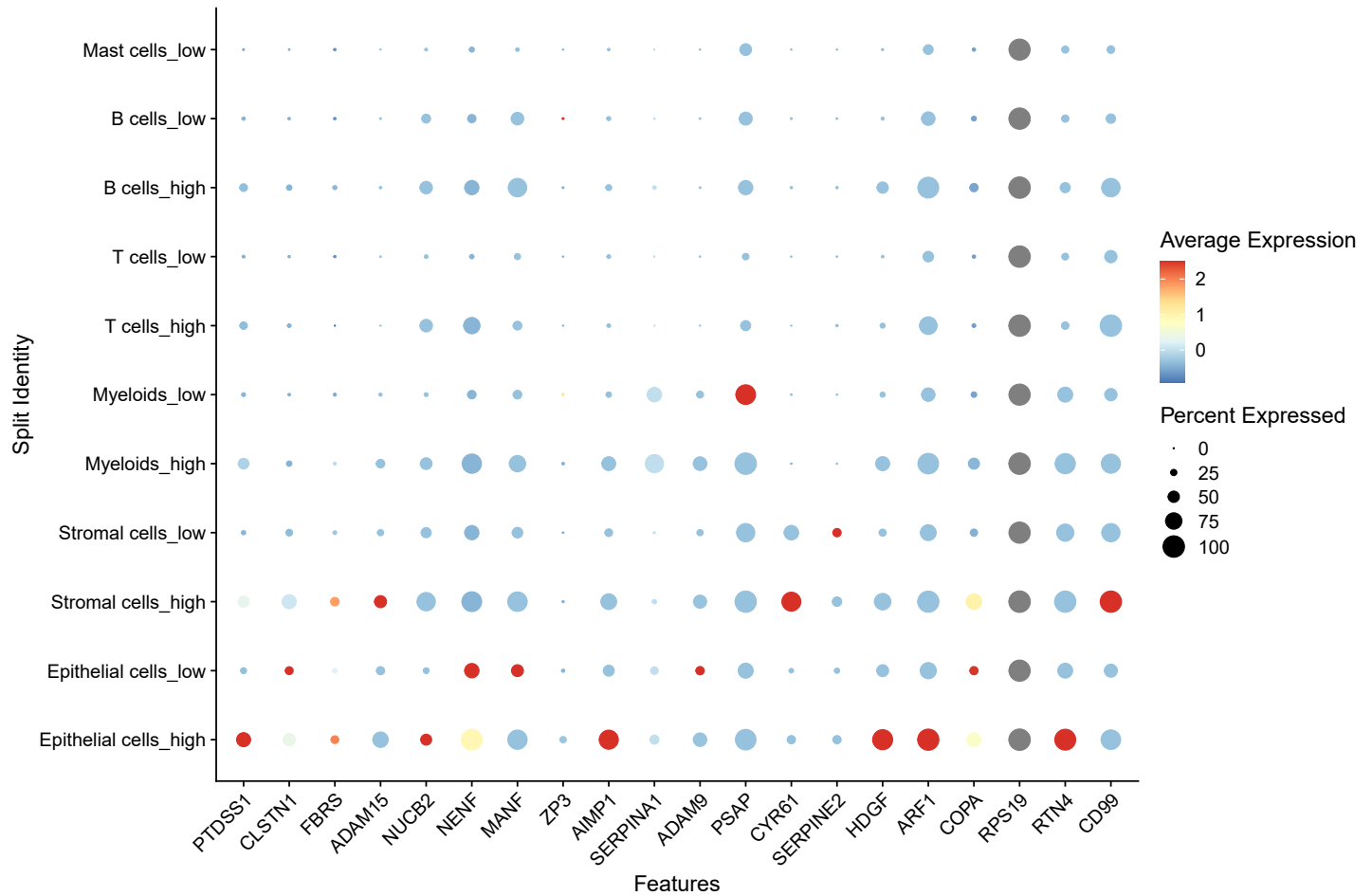

Supplement: Supplementary file 2 — Additional file 2: Figure S1.Boxplot of the expression of PAM genes in tumor and normal sample on the TCGA cohort.Heatmap of significant different PAM genes in tumor and normal sample..GO enrichment analysis for PAM genes.KEGG enrichment analysis for PAM genes.Forest plot of prognostic gene with Univariate cox regression analysis.Protein–protein interactionfor PAM-related genes.Venn diagram showing PAM genes after intersection of datasets. Figure S2. Survival prognostic analysis of each PAM gene with high and low expression using Kaplan–Meier analysis. Figure S3.Consensus clustering of 55 PAM genes matrix for k = 3 of 1224 patients in the TCGA cohort combine the GEO cohort.Determine the relevant CDF curve and Tracking plot of Consensus clustering. Figure S4.Consensus clustering of 328 PAM prognostic genes in the meta dataset.GO enrichment analysis and KEGG enrichment analysis of 328 PAM prognostic genes.Expression heatmap of 328 PAM prognostic genes in geneCluster A and B subgroups.Forest plots for univariate and multivariate cox analysis of PAMscore. Figure S5. Survival analysis of high and low score subgroups of PAMscore for different genders, different T, N, M stages and AJCC stages in CRC patients. Figure S6.RNA expression of ACAT2, SPHK1, SNED1, KPNA2, BZW2 and KIF15 in tumor and normal tissues in TCGA dataset.IHC cell staining intensity in normal and tumor tissues of the CRC cohort. Figure S7.Expression levels of marker genes in the 6 cell types.Expression levels of marker genes in high and low cell groups. [file 12935_2023_2892_MOESM2_ESM.zip › Supplementary Material 2/S7.pdf]
